# Radiation nanomedicines for cancer treatment: a scientific journey and view of the landscape

**DOI:** 10.1186/s41181-024-00266-y

**Published:** 2024-05-04

**Authors:** Raymond M. Reilly, Constantine J. Georgiou, Madeline K. Brown, Zhongli Cai

**Affiliations:** 1 Department of Pharmaceutical Sciences, University of Toronto, Toronto, ON Canada; 2 Princess Margaret Cancer Centre, Toronto, ON Canada; 3 Department of Medical Imaging, University of Toronto, Toronto, ON Canada; 4 Joint Department of Medical Imaging, University Health Network, Toronto, ON Canada; 5 Leslie Dan Faculty of Pharmacy, University of Toronto, Toronto, ON M5S 3M2 Canada

**Keywords:** Radionuclides, Cancer treatment, Gold nanoparticles, Liposomes, Block copolymer micelles, Dendrimers, Convection-enhanced delivery, Intratumoural injection, Radiosensitization, Nanomedicines

## Abstract

**Background:**

Radiation nanomedicines are nanoparticles labeled with radionuclides that emit α- or β-particles or Auger electrons for cancer treatment. We describe here our 15 years scientific journey studying locally-administered radiation nanomedicines for cancer treatment. We further present a view of the radiation nanomedicine landscape by reviewing research reported by other groups.

**Main body:**

Gold nanoparticles were studied initially for radiosensitization of breast cancer to X-radiation therapy. These nanoparticles were labeled with ^111^In to assess their biodistribution after intratumoural vs. intravenous injection. Intravenous injection was limited by high liver and spleen uptake and low tumour uptake, while intratumoural injection provided high tumour uptake but low normal tissue uptake. Further, [^111^In]In-labeled gold nanoparticles modified with trastuzumab and injected iintratumourally exhibited strong tumour growth inhibition in mice with subcutaneous HER2-positive human breast cancer xenografts. In subsequent studies, strong tumour growth inhibition in mice was achieved without normal tissue toxicity in mice with human breast cancer xenografts injected intratumourally with gold nanoparticles labeled with β-particle emitting ^177^Lu and modified with panitumumab or trastuzumab to specifically bind EGFR or HER2, respectively. A nanoparticle depot (nanodepot) was designed to incorporate and deliver radiolabeled gold nanoparticles to tumours using brachytherapy needle insertion techniques. Treatment of mice with s.c. 4T1 murine mammary carcinoma tumours with a nanodepot incorporating [^90^Y]Y-labeled gold nanoparticles inserted into one tumour arrested tumour growth and caused an abscopal growth-inhibitory effect on a distant second tumour. Convection-enhanced delivery of [^177^Lu]Lu-AuNPs to orthotopic human glioblastoma multiforme (GBM) tumours in mice arrested tumour growth without normal tissue toxicity. Other groups have explored radiation nanomedicines for cancer treatment in preclinical animal tumour xenograft models using gold nanoparticles, liposomes, block copolymer micelles, dendrimers, carbon nanotubes, cellulose nanocrystals or iron oxide nanoparticles. These nanoparticles were labeled with radionuclides emitting Auger electrons (^111^In, ^99m^Tc, ^125^I, ^103^Pd, ^193m^Pt, ^195m^Pt), β-particles (^177^Lu, ^186^Re, ^188^Re, ^90^Y, ^198^Au, ^131^I) or α-particles (^225^Ac, ^213^Bi, ^212^Pb, ^211^At, ^223^Ra). These studies employed intravenous or intratumoural injection or convection enhanced delivery. Local administration of these radiation nanomedicines was most effective and minimized normal tissue toxicity.

**Conclusions:**

Radiation nanomedicines have shown great promise for treating cancer in preclinical studies. Local intratumoural administration avoids sequestration by the liver and spleen and is most effective for treating tumours, while minimizing normal tissue toxicity.

## Background

**Fig. 1:**
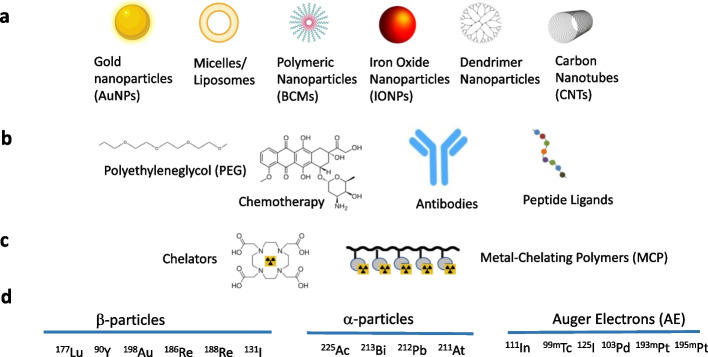
Toolbox for constructing radiation nanomedicines. **a** Various forms of nanoparticles (NPs). **b** NPs may be surface-modified with polyethyleneglycol (PEG) to minmize uptake by the liver and spleen, may incorporate chemotherapeutic agents for combined chemo-radiotherapy or may be modified with monoclonal antibodies or peptide ligands for active targeting of receptors on cancer cells. **c** NPs may be conjugated to chelators or metal-chelating polymers to complex radiometals. **d** Radiometals emitting β-particles, α-particles or Auger electrons (AEs) complexed to NPs to construct radiation nanomedicines

Radiation nanomedicine is a term that our group first used in 2015 to describe gold nanoparticles (AuNPs) conjugated to the anti-epidermal growth factor receptor (EGFR) monoclonal antibody panitumumab (Vectibix^®^, Amgen) and labeled with β-particle emitting, ^177^Lu for local treatment of EGFR-positive breast cancer (BC) (Yook et al. 2015). The term may be applied generally to describe nanoparticles (NPs) labeled with radionuclides that emit α- or β-particles or Auger electrons (AE) that are intended for cancer treatment. These radionuclides may also emit γ-photons or positrons which enable single photon emission computed tomography (SPECT) or positron emission tomography (PET), respectively. This allows assessment of the tumour and normal tissue uptake of the radiation nanomedicines by imaging, which aids in estimating the radiation absorbed doses in tumours and normal organs. However, this article does not focus on the use of radiolabeled NPs for tumour imaging, but instead on their therapeutic applications. Radiation nanomedicines may be constructed from a wide range of NPs including liposomes and polymeric micelles, graphene or carbon-based nanostructures, dendrimers and inorganic NPs, e.g. gold nanoparticles (AuNPs) (Fig. 1). They may be administered systemically [e.g. intravenous (i.v.) injection] or delivered locally [e.g. intratumoural (i.t.) injection]. Systemic administration relies on passive or active targeting to selectively deliver NPs to tumours. Passive targeting is mediated by the Enhanced Permeability and Retention (EPR) effect which is explained by increased permeability of tumour blood vessels but poor lymphatic drainage resulting in tumour accumulation of NPs (Shinde et al. 2022). Active targeting requires conjugation of biomolecules (e.g. monoclonal antibodies or peptides) to NPs to specifically bind tumour-associated receptors or other cell-surface proteins (Goddard et al. 2020). We describe here our 15 years scientific journey studying radiation nanomedicines for treatment of tumours and their preclinical evaluation. At the same time, we present a contextual view of the broader landscape of radiation nanomedicines reported by other research groups. We hope that our perspective, combined with a discussion of the landscape, will enable readers to appreciate the great promise and challenges of radiation nanomedicines for cancer treatment.

## Main text

### Scientific journey: the beginning

Our scientific journey began with studies of trastuzumab-conjugated AuNPs for X-ray therapy (XRT) radiosensitization of human epidermal growth factor receptor-2 (HER2)-positive BC (Chattopadhyay et al. 2010). We hypothesized that conjugation of trastuzumab (Herceptin, Roche) to AuNPs would target AuNPs to HER2 positive BC cells and internalize them into these cells by HER2-mediated internalization. In theory, this would maximize their radiosensitization effects in vitro combined with XRT, especially since more radiobiologically-damaging AE generated by photoelectric interaction of X-rays with AuNPs have a subcellular range. Monte Carlo (MC) simulations have shown that internalization into the cytoplasm and particularly nuclear uptake of AuNPs in cancer cells are important factors to maximize their radiosensitization properties (Cai et al. 2013; Lechtman et al. 2011). We further hypothesized that trastuzumab-conjugated AuNPs would be actively targeted to HER2-positive human BC xenografts in vivo in mice after i.v. injection, allowing studies of AuNP radiosensitization combined with XRT. In the next section, we provide a view of the landscape of the application of AuNPs as radiosensitizers combined with XRT for treatment of cancer.

### Landscape: gold nanoparticles as radiosensitizers of XRT

**Fig. 2:**
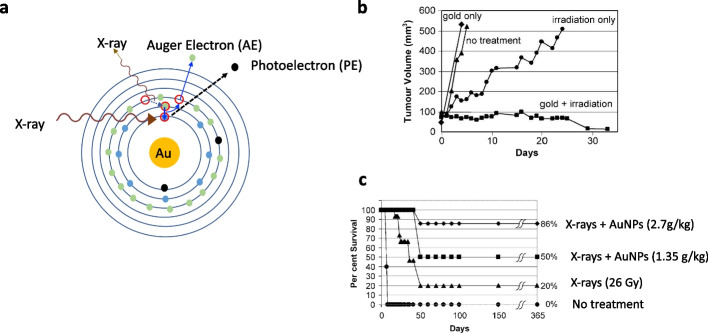
Gold nanoparticles (AuNPs) as radiosensitizers of X-radiation therapy (XRT). **a** Interaction of X-rays with an orbital shell electron in a gold atom causes release of a photoelectron (PE) creating a vacancy in the shell. This vacancy is filled by the decay of a higher shell electron, creating a subsequent vacancy that is filled by the decay of a higher shell electron, etc. in a process called an Auger cascade. Ultimately, a cascade of outer shell electrons are ejected from the atom, termed Auger electrons (AE) and X-ray photons are emitted. PE and AE have higher linear energy transfer (LET) and are thus more radiobiologically damaging than X-rays. Thus, AuNPs radiosensitize cancer cells to XRT. **b** Hainfeld et al. (2004) reported that administration of AuNPs to mice with s.c. EMT6 mammary carcinoma tumours treated with XRT arrested or decreased tumour growth, while mice receiving XRT alone only exhibited slowed tumour growth and mice treated with AuNPs alone or receiving no treatment exhibited rapid tumour growth. **c** Treatment of tumour-bearing mice with AuNPs combined with XRT improved survival compared to mice treated with XRT or receiving no treatment, and higher administered amounts of AuNPs improved survival. Reprinted (adapted) with permission from: Hainfeld JF et al. The use of gold nanoparticles to enhance radiotherapy in mice. Phys Med Biol. 2004;49:N309-15

Gold is a high atomic number (Z = 79) and dense (19.3 g/cm^3^) element that enhances the radiobiological effectiveness (RBE) of XRT, since X-rays are absorbed more efficiently by gold than soft tissues due to Compton and photoelectric interactions (Chen et al. 2020) (Fig. 2a). Interactions of X-rays with inner shell electrons in the gold atom results in the production of photoelectrons (PE) which create a vacancy in the shell, that is then filled by the decay of an electron from a higher shell, creating a subsequent vacancy with a X-ray photon emission or two subsequent vacancies with the emission of an electron called an AE. These vacancies are filled by the decay of higher shell electrons, and so on, in what is termed an Auger cascade. Ultimately, the Auger cascade results in the ejection of a series of outer shell AE, or emission of low energy X-rays. Both PE and AE are high linear energy transfer (LET) forms of radiation that are more radiobiologically damaging than low LET X-rays. PE have higher energy than AE and a longer range in tissue (e.g. ~ 100 μm for a 100 keV PE), while AE have much lower energy and penetrate much shorter distances (e.g. < 10 nm) (Hainfeld et al. 2008). The most widely studied form of gold for radiosensitization are AuNPs, which are typically spherical gold particles with a diameter < 100 nm (Chen et al. 2020). In addition to generating high LET, several biological mechanisms have been proposed to explain AuNP radiosensitization by PE and AE including: (i) increased production of reactive oxygen species (ROS), (ii) disruption of the cell cycle, resulting in accumulation of cells in the more radiosensitive G2/M-phase, (iii) interference in DNA repair, and (iv) a bystander effect that extends the effects of X-radiation to non-irradiated cells (Her et al. 2017; Rosa et al. 2017).

Great interest in AuNP radiosensitization was sparked by a landmark report in 2004 by Hainfeld et al. (2004) who studied XRT of subcutaneous (s.c.) EMT-6 murine mammary carcinoma tumours in Balb/c mice using 250 kVp X-rays with or without intravenous (i.v.) injection of AuNPs (1.9 nm). Tumour growth arrest and shrinkage was achieved in mice treated with XRT combined with AuNPs, but tumour growth was only slowed in mice that received XRT alone, and tumours grew rapidly in mice treated with AuNPs without XRT and in untreated mice (Fig. 2b). Moreover, AuNP radiosensitization prolonged the survival of XRT treated mice, dependent on the amount of AuNPs administered (Fig. 2c). Despite these encouraging results, a limitation was the high amount of gold administered to mice (1.35 g/kg or 2.7 g/kg) that was required to achieve sufficient concentrations for radiosensitization (0.5–1% of tumour weight equivalent to 10 mg of gold per g of tumour) (Hainfeld et al. 2008; Roeske et al. 2007). These amounts in some cases were just slightly lower than the lethal dose-50% (LD_50_ ~ 3.2 g/kg). Nonetheless, this study demonstrated proof-of-principle and led to many investigations of AuNPs as radiosensitizers, including Monte Carlo (MC) dose simulations, studies of their radiosensitizing effects in vitro by decreasing the clonogenic survival (CS) of cancer cells exposed to XRT, and in vivo studies of tumour growth inhibition combined with XRT (Dheyab et al. 2023; Hainfeld et al. 2008; Her et al. 2017). The radiosensitization properties of AuNPs are defined by a dose-enhancement factor (DEF) which is the radiobiological effect (e.g. decreased CS) for XRT combined with AuNPs vs. XRT alone. DEFs for cancer cells exposed in vitro to XRT combined with AuNPs vs. XRT alone have ranged from 1.1 to 1.9, but most were 1.1–1.4 (Her et al. 2017). There have been fewer studies of AuNP radiosensitization in vivo, but in addition to the study by Hainfeld et al. (2004), AuNP radiosensitization has been reported in several other mouse tumour models (Her et al. 2017).

MC modeling revealed that several factors control the dose enhancement by AuNPs including X-ray energy, concentration and location of AuNPs in cells and the size of AuNPs. The strongest absorption of X-ray energy occurs at the K-edge (80.7 keV), L-edges (11.9–14.4 keV) or M-edges (2.2–3.4 keV) of gold (Hainfeld et al. 2008). These edges represent the minimum energy of an incident X-ray needed to eject an electron from a particular shell. However, radiation treatment of cancer is most often delivered by much higher energy (megavoltage) photons that are not optimally absorbed by gold. Lechtman et al. (2011) predicted that 300-times greater gold concentration in tumours (1560–1760 mg/g) would be required to achieve a twofold increase in the absorbed dose (i.e. DEF = 2) for 6 MV photons than for an ^125^I brachytherapy source with lower average energy = 27 keV (5.3–6.3 mg/g). These gold concentrations are very high, and likely not feasible for radiosensitization with high energy photons. The size of AuNPs affects radiosensitization because a proportion of low energy AE produced by photoelectric interaction with X-rays are absorbed by the NPs themselves, and this increases for larger AuNPs. In addition, the AEs emitted have a range < 1 μm, and thus nuclear localization of AuNPs may be needed to maximize DNA damage (Lechtman et al. 2011). Dosimetry modeling reveals that nuclear uptake maximizes the DEF (Lechtman et al. 2013). Cai et al. (2013) defined a new term: Nuclear Dose Enhancement Factor (NDEF) which predicts the radiosensitization effects of AuNPs. By MC simulation the effect of various factors on NDEF were studied. These included different photon sources [monoenergetic X-rays (10–100 keV), X-ray beam (100 kVp) or ^125^I or ^103^Pd brachytherapy seeds], different numbers or diameter (5, 30 or 50 nm) of AuNPs located on the cell surface, in the cytoplasm or nucleus of human BC cells or in the extracellular space as well as different cell geometries (single cell, monolayer or cell cluster). NDEFs were greatest for AuNPs in the nucleus, and using X-rays with energy of 15 or 40 keV. The NDEF estimated by monolayer cell geometry was most correlated with the experimentally measured AuNP radiosensitization in CS assays. The cellular concentration of gold needed to achieve an NDEF = 2 for X-rays combined with AuNPs versus X-rays alone for 30 nm AuNPs was 5.1 mg/g, when AuNPs were placed in the nucleus compared to 10 mg/g in the cytoplasm or the cell surface. These model-predicted concentrations agree with those used by Hainfeld et al. in mice with EMT-6 tumours for XRT radiosensitization (Hainfeld et al. 2004) (10 mg gold/g).

### Journey: HER2-targeted AuNPs for XRT radiosensitization

**Fig. 3:**
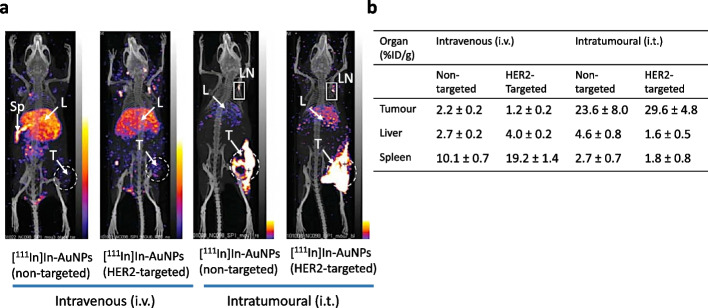
**a** SPECT/CT images at 48 h post-injection (p.i.) of non-targeted or HER2-targeted [^111^In]In-labeled AuNPs in mice with s.c. MDA-MB-361 human breast cancer (BC) xenografts after intravenous (i.v.) or intratumoural (i.t.) injection. High liver (L) and/or spleen (Sp) sequestration but low tumour (T) uptake was observed for i.v. injection. High tumour uptake but very low liver (L) uptake and no spleen accumulation were observed after i.t. injection. Uptake in an axillary lymph node (LN) is also seen after i.t. injection. **b** Biodistribution studies revealed high liver and spleen uptake of i.v. injected [^111^In]In-labeled AuNPs and low tumour uptake, while i.t. injected [^111^In]In-labeled AuNPs had much greater tumour uptake and lower liver and spleen uptake. Reprinted (adapted) with permission from: Chattopadhyay, N. et al. Role of antibody-mediated tumor targeting and route of administration in nanoparticle tumor accumulation in vivo. Mol. Pharm. 2012;9:2168–2179. Copyright 2012 American Chemical Society

To explore AuNP radiosensitization of XRT of HER2-positive BC, we conjugated AuNPs (30 nm) to trastuzumab through a 5 kDa cross-linker: orthopyridyldisulphide polyethylene glycol-N-hydroxysuccinimide valerate (OPSS-PEG_5K_-SVA). Trastuzumab-PEG-OPSS were linked to AuNPs by a gold-thiol bond formed by reaction with the OPSS group (Chattopadhyay et al. 2010). Trastuzumab was modified with ~ 7 OPSS-PEG_5K_ chains per molecule and each AuNP was conjugated to ~ 14 trastuzumab-PEG_5K_-OPSS. AuNPs were then surface-coated with 2 kDa PEG_2K_-SH chains to prevent aggregation. These modifications increased the hydrodynamic diameter of the AuNPs to ~ 60 nm. Darkfield microscopy showed HER2-specific binding, internalization and perinuclear localization of trastuzumab-AuNPs by SK-BR-3 human BC cells. Exposure of SK-BR-3 cells to 300 kVp X-rays combined with HER2-targeted AuNPs increased DNA DSBs by 5.5-fold, while non-targeted AuNPs increased DNA DSBs by 3.3-fold compared to XRT alone. DNA damage was determined by immunofluorescence microscopy for γ-H2AX, a phosphorylated form of histone-2A that accumulates at sites of unrepaired DNA DSBs (Mah et al. 2011). Subsequently, we investigated HER2-targeted AuNPs combined with 100 kVp X-rays for decreasing the CS of MDA-MB-361 human BC cells in vitro and for inhibiting the growth of s.c. MDA-MB-361 tumour xenografts in vivo in athymic mice (Chattopadhyay et al. 2013). The dose required to decrease the CS of MDA-MB-361 cells to 0.10 (D_10_) was 7.6 Gy for X-rays, but was 5.9 Gy and 4.8 Gy for XRT combined with non-targeted or HER2-targeted AuNPs, respectively. These D_10_ values corresponded to a DEF = 1.3 or 1.6 for non-targeted or HER2-targeted AuNPs, respectively. HER2-targeted AuNPs inflicted more DNA DSBs than non-targeted AuNPs combined with XRT. To study the radiosensitizing effects of AuNPs in vivo, we employed i.t. injection of HER2-targeted AuNPs in mice with s.c. MDA-MB-361 tumours to achieve a sufficient concentration of gold for radiosensitization (5–10 mg/g) (Cai et al. 2013). The selection of i.t. injection was informed by an earlier imaging and biodistribution study that showed that i.v. injected HER2-targeted AuNPs labeled with ^111^In exhibited very low tumour uptake at 48 h post-injection (p.i.) [1.2 percent injected dose/g (%ID/g)] and high uptake in the spleen (19.2% ID/g), liver (2.7% ID/g) and kidneys (2.3% ID/g) (Chattopadhyay et al. 2012) (Fig. 3). In contrast, i.t. injection of ^111^In-labeled HER2-targeted AuNPs resulted in 25-fold greater tumour uptake (29.6% ID/g) and decreased spleen uptake by 11-fold (1.8% ID/g) while moderately reducing uptake in the liver (1.6% ID/g) and kidneys (1.5% ID/g). Most NPs injected i.v. are recognized and captured by the mononuclear phagocyte system (MPS) which includes the lymph nodes, spleen and liver. Despite shielding of NPs by PEG, spleen and liver uptake remain a major obstacle to their delivery to tumours (Mills et al. 2022). Intratumoural injection of HER2-targeted AuNPs minimized spleen and liver uptake and achieved a tumour concentration of gold (4.8 mg/g) that was expected to be sufficient to radiosensitize tumours to XRT (Cai et al. 2013). Moreover, this tumour concentration of gold was achieved by i.t. injection of only 0.8 mg of AuNPs (0.04 g/kg) which was 33-fold lower than that injected i.v. in mice with EMT-6 tumours for XRT radiosensitization (2.7 g/kg) by Hainfeld et al. (2004), improving the safety profile of AuNP radiosensitization. Treatment of mice with s.c. MDA-MB-361 tumours with HER2-targeted AuNPs combined with 11 Gy of XRT decreased tumour volume by ~ twofold over 5 weeks, while tumours treated with only XRT increased in size. No normal tissue toxicity was found in mice treated with XRT alone or combined with HER2-targeted AuNPs, assessed by monitoring body weight and hematology and blood biochemistry analyses.

### Journey: radiation nanomedicine for HER2-positive BC

**Fig. 4:**
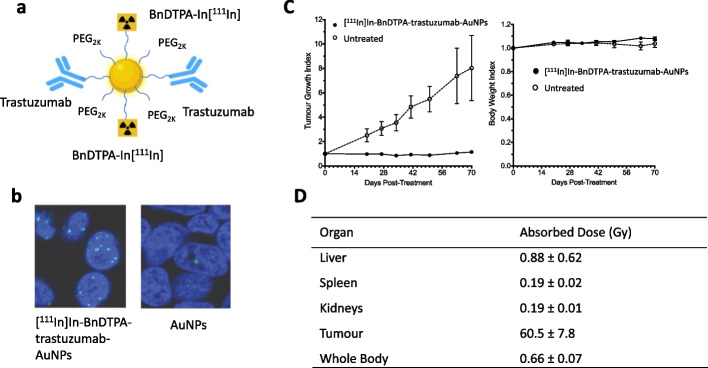
**a** Auger electron (AE)-emitting radiation nanomedicine composed of AuNPs modified with PEG linked to BnDTPA complexed to ^111^In and to trastuzumab for binding HER2 on breast cancer (BC) cells. **b** Immunofluorescence staining for γ-H2AX showed that emission of AE caused DNA double-strand breaks (DSBs; bright foci) in the nucleus (blue) of HER2-positive MDA-MB-361 human BC cells, while cells exposed to unlabeled AuNPs did not show DNA DSBs. **c** Intratumoural (i.t.) injection of [^111^In]In-BnDTPA-trastuzumab-AuNPs (10 MBq; 2.6 × 10^12^ AuNPs) arrested the growth of MDA-MB-361 tumours in mice, while untreated mice exhibited rapid tumour growth (left panel). There was no decrease in body weight (right panel) indicating no general normal tissue toxicity. **d** Absorbed doses in the tumour and selected normal organs from i.t. injection of this radiation nanomedicine. The tumour dose was high (> 60 Gy) while normal organ doses were low (< 1 Gy). Reprinted (adapted) with permission from: Cai, Z. et al. ^111^In-labeled trastuzumab-modified gold nanoparticles are cytotoxic in vitro to HER2-positive breast cancer cells and arrest tumor growth in vivo in athymic mice after intratumoral injection. Nucl. Med. Biol. 2016;43:818–826

Trastuzumab-AuNPs labeled with ^111^In were used in the previously described XRT radiosensitization study to compare their tumour and normal tissue uptake after i.v. or i.t. injection in mice with s.c. MDA-MB-361 human BC xenografts by SPECT and by ex vivo γ-counting of tissues (Chattopadhyay et al. 2012). ^111^In (t_1/2_ = 2.8 d) decays by electron capture (EC) emitting two γ-photons [Eγ = 171 keV (90%) and Eγ = 245 keV (94%)] that allow SPECT and 7.4 AEs (average energy = 0.9 keV) and 0.2 internal conversion electrons (CEs) per decay (average energy = 27.9 keV) that may be used for radiotherapeutic purposes (Ku et al. 2019). [^111^In]In-BnDTPA-trastuzumab-AuNPs (Fig. 4a) were synthesized by conjugation of AuNPs to [^111^In]In-Bn-DTPA-PEG_2K_-SH (Chattopadhyay et al. 2012). [^111^In]In-Bn-DTPA-PEG_2K_-SH was synthesized by reaction of PEG_2K_-NH_2_ with S-2-(4-Isothiocyanatobenzyl)-diethylenetriamine pentaacetic acid (p-SCN-Bn-DTPA) then labeled with ^111^In in sodium acetate buffer, pH 6.0. Based on our observation that i.t. injected [^111^In]In-Bn-DTPA-PEG_2K_-trastuzumab-AuNPs were strongly retained in s.c. MDA-MB-361 tumours in athymic mice and exhibited low uptake in normal tissues, we hypothesized that the AE and CE emissions of ^111^In may be effective for inhibiting tumour growth at amounts that would not cause toxicity to normal tissues. This was the first time in our journey that we considered the idea of a radiation nanomedicine administered locally for treatment of tumours. We tested this idea by first determining the effectiveness of [^111^In]In-Bn-DTPA-PEG_2K_-trastuzumab-AuNPs for decreasing the CS of HER2-positive SK-BR-3 and MDA-MB-361 human BC cells in vitro, and for inhibiting the growth of s.c. MDA-MB-361 tumours in vivo in mice (Cai et al. 2016). [^111^In]In-Bn-DTPA-PEG_2K_-trastuzumab-AuNPs were specifically bound and internalized by SK-BR-3 and MDA-MB-361 cells in vitro and were transported to a perinuclear location. Nuclear localization of these AuNPs may be mediated by a nuclear translocation sequence (NLS) present in HER2 (Chen et al. 2005). The emission of AEs and CEs by ^111^In in close proximity to the nucleus caused DNA DSBs (Fig. 4b) that decreased the CS of both SK-BR-3 and MDA-MB-361 cells. Most importantly, i.t. injection of 10 MBq of [^111^In]In-Bn-DTPA-PEG_2K_-trastuzumab-AuNPs (2.6 × 10^12^ AuNPs) in mice with s.c. MDA-MB-361 human BC xenografts arrested tumour growth with no decrease in body weight which indicated no general toxicity (Fig. 4c). The absorbed dose in these tumours was high (60.5 Gy), while normal organ doses were low (< 0.9 Gy) and the whole body dose was 0.66 Gy (Fig. 4d). In mice treated with [^111^In]In-Bn-DTPA-PEG_2K_-trastuzumab-AuNPs, there were no significant changes in complete blood cell counts (CBC) and serum biochemistry [e.g. alanine aminotransferase (ALT) or creatinine (Cr)] compared to normal saline-treated mice, indicating no normal tissue toxicity. These results greatly encouraged us to continue investigations of a radiation nanomedicine strategy for local treatment of BC and other tumours. In the next section, we discuss the landscape of various forms of NPs labeled with AE-emitting radionuclides that have been studied for cancer treatment.

### Landscape: NPs labeled with AE-emitting radionuclides

#### Indium-111

Song et al. (2016) conjugated 14 nm AuNPs to [^111^In]In-DTPA-human epidermal growth factor (^111^In]In-DTPA-EGF) by a gold-thiol bond formed with the disulfides in EGF. Up to 78 DTPA-hEGF were linked to one AuNP. [^111^In]In-DTPA-EGF-AuNPs were specifically bound and internalized by EGFR-overexpressing MDA-MB-468 human BC cells and confocal fluorescence microscopy with AuNPs linked to Cy3-EGF showed perinuclear localization. Nuclear localization may be mediated by a NLS present in the EGFR that binds to importins responsible for nuclear importation of proteins (Hsu and Hung 2007). Emission of AEs by [^111^In]In-DTPA-EGF-AuNPs decreased the CS in vitro of MDA-MB-468 cells, but did not decrease the CS of MCF-7 human BC cells that have low EGFR expression. Subsequently, these [^111^In]In-DTPA-EGF-AuNPs were surface-coated with PEG-SH and compared to non-PEGylated forms (Song et al. 2017). PEGylation increased the hydrodynamic diameter of [^111^In]In-DTPA-EGF-AuNPs from 18 to 32 nm and decreased surface charge (zeta potential) from − 24 to − 9.6 mV. PEGylated [^111^In]In-DTPA-EGF-AuNPs were internalized by MDA-MB-468 and MDA-MB-231/H2N human BC cells and Cy3-EGF-AuNPs showed perinuclear localization. Unfortunately, there was high liver and kidney uptake and low tumour accumulation (0.2% ID/g) in mice with s.c. MDA-MB-468 or MDA-MB-231/H2N tumours at 72 h post-i.v. injection of non-PEGylated [^111^In]In-DTPA-EGF-AuNPs. PEGylation reduced liver uptake by twofold and increased tumour uptake by 14-fold (2.8% ID/g). Co-administration of excess EGF to block EGFR on normal tissues further decreased uptake in liver, kidneys and spleen but decreased tumour uptake (1.4% ID/g). An interesting method of labeling AuNPs with ^111^In by incorporating [^111^In]InCl_3_ into the synthesis of AuNPs was reported by Ng et al. (2014). These [^111^In]In-AuNPs were further modified with arginine-glycine-aspartic acid (RGD) peptides to target α_v_β_3_ integrins displayed on M21 melanoma or U87-MG glioblastoma multiforme (GBM) cells. Unfortunately, tumour uptake of [^111^In]In-RGD-AuNPs at 4 h post i.v. injection in mice with s.c. M21 or U87-MG xenografts was low (0.5–1% ID/g) while liver and spleen uptake were high (18–30% ID/g and 8–12% ID/g, respectively).

Block copolymer micelles (BCMs) are NPs composed of amphiphilic copolymers that form micellar structures containing a hydrophobic core in which drugs may be loaded, surrounded by a hydrophilic corona that may be modified with targeting ligands (Fonge et al. 2012). Fonge et al. (2010) constructed EGFR-targeted BCMs (13–15 nm) from MePEG_2500_-*b*-PCL_1200_ and [^111^In]In-DTPA-PEG_3000_-*b*-PCL_1600_ copolymers and evaluated their uptake in MDA-MB-468, MDA-MB-231 and MCF-7 human BC cells with high, moderate or low EGFR density, respectively. The effect of exposure of MDA-MB-468 or MCF7 cells in vitro to EGFR-targeted [^111^In]In-DTPA-PEG-EGF BCMs or non-targeted [^111^In]In-DTPA-PEG BCMs was assessed in CS assays. Uptake in MDA-MB-468, MDA-MB-231 or MCF-7 cells was dependent on the EGFR expression level, but most activity remained on the cell surface, with a small amount of internalization into the cytoplasm and nucleus. Nonetheless, [^111^In]In-DTPA-PEG-EGF BCMs strongly decreased the CS of MDA-MB-468 cells that have high EGFR expression, but not MCF-7 cells with low EGFR expression. Non-targeted [^111^In]In-DTPA-PEG BCMs were not cytotoxic. Interestingly, a comparison with [^111^In]In-DTPA-EGF revealed more efficient binding of [^111^In]In-DTPA-EGF than [^111^In]In-DTPA-PEG-EGF BCMs to MDA-MB-468 cells and higher nuclear importation, resulting in greater cytotoxicity. [^111^In]In-DTPA-EGF is a radiopeptide that has been previously reported as an AE-emitting radiotherapeutic agent for EGFR-positive BC (Reilly et al. 2000). Hoang et al. (2012) constructed HER2-targeted BCMs composed of MePEG-*b*-PCL, [^111^In]In-DTPA-PEG-*b*-PCL and NLS_2_-trastuzumab-Fab-PEG-*b*-PCL copolymers. The size of these BCMs was 35 nm and their zeta potential was + 2.0 mV. NLS_2_-trastuzumab-Fab-PEG-*b*-PCL consisted of trastuzumab Fab linked to NLS peptides (CGYGPKKKKRKVGG) conjugated to PEG-*b*-PCL. These BCMs incorporated methotrexate (MTX) into the core, which was intended as a radiosensitizer for AEs emitted by ^111^In, since MTX inhibits DNA repair (Costantini et al. 2010). These BCMs were specifically bound and internalized by HER2-positive SK-BR-3 and MDA-MB-361 human BC cells in vitro and the NLS_2_-trastuzumab-Fab-PEG-*b*-PCL copolymer mediated nuclear localization. The CS of SK-BR-3 and MDA-MB-361 cells were greatly decreased by the emission of AEs by ^111^In, and this was amplified by incorporating MTX into these BCMs. These HER2-targeted BCMs were not studied in vivo, but the tumour and normal tissue uptake of non-targeted BCMs composed of MePEG-*b*-PCL and [^111^In]In-DTPA-PEG-*b*-PCL copolymers has been studied after i.v. injection in mice with s.c. MDA-MB-231 tumour xenografts (Hoang et al. 2009). Similar to other NPs administered by i.v. injection, there was high liver (13% ID/g) and spleen (22% ID/g) sequestration but tumour uptake was relatively high (9% ID/g). Therapeutic effectiveness and toxicity in vivo were not assessed.

Other forms of NPs labeled with ^111^In have been studied as radiation nanomedicines. Chan et al. (2013) reported that G4 polyamidoamine (PAMAM) dendrimers conjugated to trastuzumab and multiple DTPA for high specific activity (SA) labeling with ^111^In were internalized into the cytoplasm and nucleus of HER2-positive SK-BR-3 cells. Emission of AEs caused DNA DSBs which decreased the CS of these cells. Thomas et al. (2019) conjugated [^111^In]In-DTPA-EGF to liposomes (140 nm; zeta potential = − 30 mV) that also incorporated doxorubicin for combined chemoradiotherapy of EGFR-overexpressing BC. [^111^In]In-DTPA-EGF-Dox-liposomes were bound and internalized by EGFR-overexpressing MDA-MB-468 human BC cells and localized in the nucleus. Emission of AEs by ^111^In caused DNA DSBs which were amplified by the DNA damaging properties of doxorubicin, and strongly decreased the CS of MDA-MB-468 cells. One interesting aspect of this study was that focused ultrasound was applied to s.c. MDA-MB-468 tumours to improve the tumour penetration in vivo of [^111^In]In-DTPA-EGF-Dox-liposomes following i.v. injection in mice. Nonetheless, tumour uptake remained very low (0.3% ID/g) at 48 h p.i. and liver and kidney uptake were high (33% ID/g and 11% ID/g, respectively), again demonstrating the challenges associated with i.v. administration of NPs.

#### Technetium-99 m

Jimenez-Mancilla et al. (2013) conjugated AuNPs (5 nm) to Tat (transactivation of transcription) peptides linked through a GCGC peptide spacer to bombesin peptides to target gastrin-releasing peptide (GRP) receptors on prostate cancer cells. These AuNPs were dual-labeled with the AE-emitter, ^99m^Tc and β-particle-emitter, ^177^Lu by binding of [^99m^Tc]Tc-hydrazinonicotinyl-Tyr^3^-octreotide ([^99m^Tc]Tc-HYNIC-TOC) and [^177^Lu]Lu-DOTA-GGC peptides to the AuNPs. Tat peptides promote cell penetration and harbour a NLS that enables nuclear importation (Costantini et al. 2008), where the AEs emitted by ^99m^Tc are most damaging to DNA. These radiation nanomedicines were cytotoxic against PC-3 human prostate cancer cells in vitro, reducing their proliferation by > 95%. Interestingly, although the absorbed dose in PC-3 cells from ^99m^Tc was 14-fold lower than ^177^Lu (4 Gy/Bq vs. 55 Gy/Bq), dual-labeled AuNPs were eight-fold more cytotoxic than ^177^Lu-labeled AuNPs alone, which was attributed to the greater radiobiological effectiveness of the AEs vs. β-particles. Intratumoural (i.t.) injection of dual-labeled AuNPs in mice with s.c. PC-3 tumours resulted in high tumour uptake (58% ID/g) that was retained up to 24 h p.i. (Jimenez-Mancilla et al. 2012). The absorbed dose in the tumours was 7.9 Gy/MBq but the estimated dose in the nucleus of tumour cells was 0.53 Gy/MBq, demonstrating the potential of dual labeled radiation nanomedicines for treating tumours exploiting both AEs and β-particles.

#### Iodine-125, Palladium-103 and Platinum-193m/195m

Zhang et al. (2019) synthesized spherical AuNPs or gold nanorods (AuNRs) conjugated to RGD peptides to target α_v_β_3_ integrins and loaded with cisplatin (cis-Pt) for combined chemoradiation therapy targeting the tumour vasculature. Non-radiolabeled RGD-[^127^I]I-cis-Pt-AuNRs exhibited slightly higher uptake than RGD-[^127^I]I-cis-Pt-AuNPs in H1299 human non-small cell lung cancer cells in vitro, which yielded greater radiosensitization when these cells were exposed to XRT (4 Gy). RGD-[^125^I]I-cis-Pt-AuNPs and RGD-[^125^I]I-cis-Pt-AuNRs were accumulated in s.c. HI299 tumors in vivo in mice demonstrated by SPECT/CT, but higher uptake was found for [^125^I]I-cis-Pt-AuNRs. There was high uptake in the liver and spleen. Non-radiolabeled RGD-[^127^I]I-cis-Pt-AuNPs and RGD-[^127^I]I-cis-Pt-AuNRs were studied for chemoradiation therapy in mice with H1299 tumors treated with XRT (6 Gy). RGD-[^127^I]I-cis-Pt-AuNRs were most effective combined with XRT for inhibiting tumor growth. Chemoradiation therapy with RGD-[^127^I]I-cis-Pt-AuNPs or RGD-[^127^I]I-cis-Pt-AuNRs was more effective than XRT alone. This study did not examine the cytotoxic effects of the AE emissions from ^125^I-labeled radiation nanomedicines, but this may be interesting to study in the future, since ^125^I emits a high number of AE/decay (23.0) and total AE energy/decay (12 keV) (Ku et al. 2019). ^193m^Pt and ^195m^Pt are also attractive AE-emitting radionuclides, which emit 27.4 AE/decay and 36.6 AE/decay, respectively (total energy = 10.9 keV and 23.1 keV, respectively) (Ku et al. 2019). Wawrowicz et al. (2021) synthesized AuNPs (~ 37 nm) incorporating a platinum shell that were then PEGylated and modified with trastuzumab to target HER2-positive BC cells. Trastuzumab-PEG-[^195^Pt]Pt-AuNPs incorporating stable ^195^Pt were bound, internalized and transported to a perinuclear location in HER2-positive SK-OV-3 cells in vitro. The internalization and nuclear transport of trastuzumab-PEG-[^195^Pt]Pt-AuNPs suggests that these agents incorporating the AE-emitters ^193^Pt or ^195m^Pt may be cytotoxic to HER2-positive BC cells and provide useful radiation nanomedicines for treating BC overexpressing HER2, but further studies are needed to explore this approach.

### Journey: ^177^Lu-labeled radiation nanomedicines

A limitation of AEs is their subcellular range, which is only a few nm to < 1 μm (Ku et al. 2019). This requires binding and internalization into cancer cells, and ideally routing to the nucleus to maximize lethal DNA DSBs (Ku et al. 2019). Thus, in the next step on our journey, we explored AuNPs labeled with ^177^Lu (t_1/2_ = 6.7 d) which emits longer range (maximum 2 mm) β-particles, that are able to irradiate and kill tumour cells even without binding to, internalizing in tumour cells or translocating to the cell nucleus. ^177^Lu emits moderate energy β-particles [Eβ_max_ = 0.50 MeV (78.6%), 0.38 MeV (9.1%), 0.18 MeV (12.2%)] and a γ-photon (Eγ = 208 keV (11%)] that allows SPECT imaging. EGFR-targeted radiation nanomedicines were constructed by conjugating OPSS-PEG_5K_-DOTA-^177^Lu and OPSS-PEG_5K_-panitumumab to AuNPs (30 nm). Non-targeted AuNPs were synthesized by conjugating only OPSS-PEG_5K_-DOTA-^177^Lu to AuNPs (Yook et al. 2015). DOTA [2,2′,2′′,2′′′-(1,4,7,10-tetraazacyclododecane-1,4,7,10-tetrayl)tetraacetic acid] is commonly used to chelate ^177^Lu and other radiometals (e.g. ^90^Y, ^225^Ac, ^111^In, or ^64^Cu) (Sneddon and Cornelissen 2021). Darkfield microscopy revealed binding of EGFR-targeted but not non-targeted AuNPs to EGFR-positive MDA-MB-468 human BC cells. Confocal immunofluorescence microscopy probing for panitumumab with AlexaFluor-488 anti-human IgG showed internalization of EGFR-targeted-AuNPs into these cells. Binding and internalization of [^177^Lu]Lu-DOTA-PEG_5K_-panitumumab-AuNPs were EGFR-dependent determined by subcellular fractionation studies of MDA-MB-468, MDA-MB-231 and MCF-7 cells with high, intermediate or low EGFR expression, respectively. Absorbed doses in the nucleus of MDA-MB-468 cells treated in vitro with 4.5 MBq (6 × 10^11^ AuNPs) of EGFR-targeted [^177^Lu]Lu-DOTA-PEG_5K_-panitumumab-AuNPs (73.2 Gy) were 13-fold greater than non-targeted [^177^Lu]Lu-DOTA-PEG_5K_-AuNPs (5.6 Gy). Exposure of MDA-MB-468 cells in vitro to [^177^Lu]Lu-DOTA-PEG_5K_-panitumumab-AuNPs reduced their CS to < 0.1%. Non-targeted [^177^Lu]Lu-DOTA-PEG-AuNPs were also cytotoxic in vitro, due to the cross-fire effect from the 2 mm range β-particles, but these were > 100-fold less cytotoxic (CS = 8.4%) than EGFR-targeted [^177^Lu]Lu-DOTA-PEG_5K_-panitumumab-AuNPs.

**Fig. 5:**
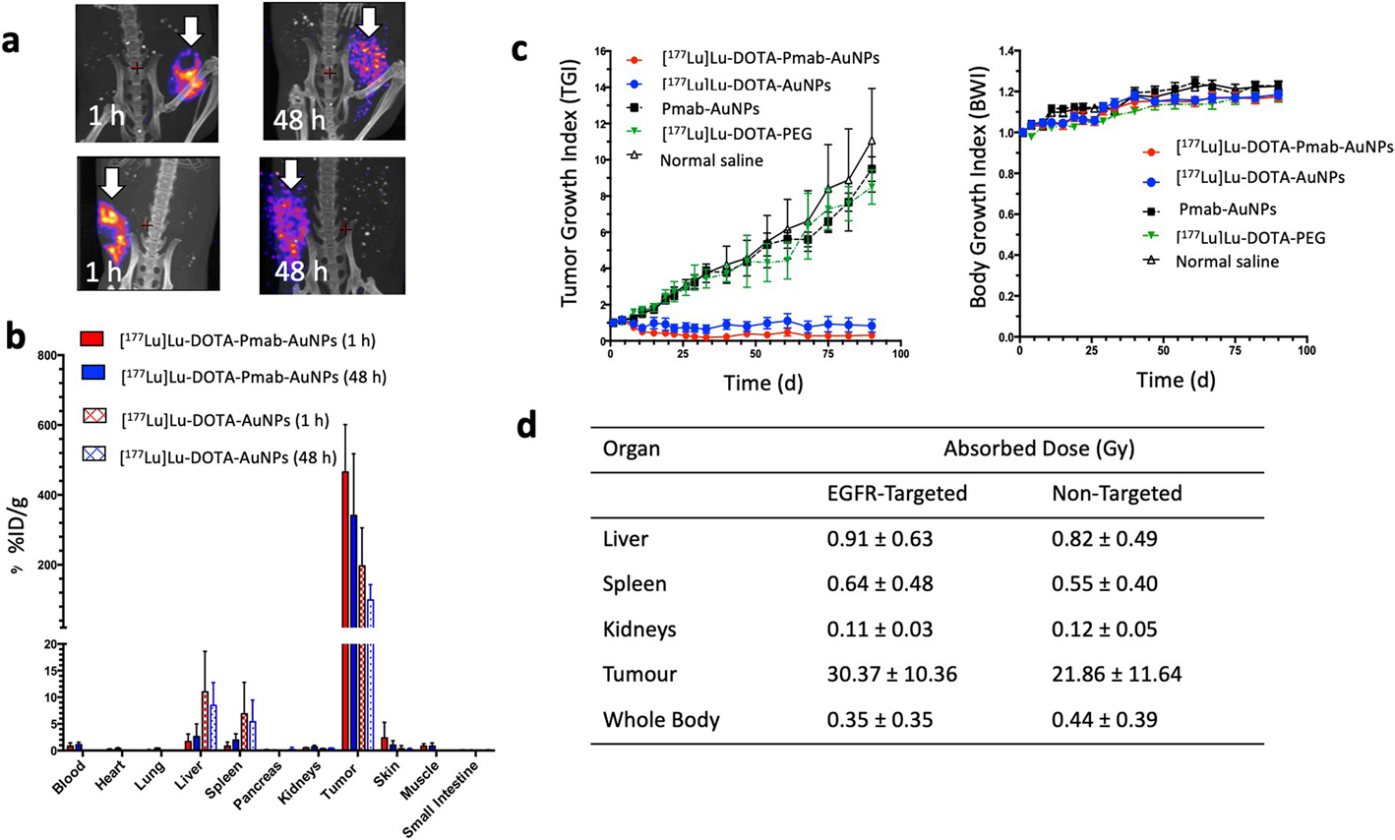
**a** SPECT/CT images of mice at 1 h and 48 h after intratumoural (i.t.) injection of EGFR-targeted [^177^Lu]Lu-DOTA-panitumumab (Pmab)-AuNPs (top panels) or non-targeted [^177^Lu]Lu-DOTA-AuNPs (bottom panels) in athymic mice with s.c. human MDA-MB-468 human breast cancer (BC) xenografts. **b** Tumour and normal tissue biodistribution at 1 h and 48 h after i.t. injection of [^177^Lu]Lu-DOTA-panitumumab-AuNPs or [^177^Lu]Lu-DOTA-AuNPs. **c** Effect of i.t. injection of EGFR-targeted or non-targeted radiation nanomedicines or control treatments on the growth of s.c. MDA-MB-468 human BC xenografts in athymic mice (left panel). Effect of radiation nanomedicines or control treatments on body weight (right panel). **d** Absorbed doses in the tumour and normal organs for i.t. injection of EGFR-targeted or non-targeted radiation nanomedicines. Reprinted (adapted) with permission from: Yook, S. et al. Intratumorally injected ^177^Lu-labeled gold nanoparticles: gold nanoseed brachytherapy with application for neoadjuvant treatment of locally advanced breast cancer. J. Nucl. Med. 2016;57:936–942

We subsequently compared [^177^Lu]Lu-DOTA-PEG_5K_-panitumumab-AuNPs and [^177^Lu]Lu-DOTA-PEG_5K_-AuNPs injected i.t. for treatment of s.c. MDA-MB-468 tumours in mice (Yook et al. 2016a). Imaging (Fig. 5a) and biodistribution studies (Fig. 5b) at 48 h p.i. revealed high uptake and retention in the tumour, which was twofold greater for EGFR-targeted AuNPs (197% ID/g) than non-targeted AuNPs (99% ID/g). Normal tissue uptake was very low (< 0.5% ID/g) for EGFR-targeted and non-targeted AuNPs, except for the liver (8.5–11% ID/g) and spleen (5.4–6.9% ID/g). Both [^177^Lu]Lu-DOTA-PEG_4K_(panitumumab-PEG_5K_)-AuNPs and [^177^Lu]Lu-DOTA-PEG_4K_-AuNPs injected i.t. (4.5 MBq; 6 × 10^11^ AuNPs) arrested the growth of MDA-MB-468 tumours without decreasing body weight (Fig. 5c) and significantly prolonged the survival of tumour-bearing mice. No normal tissue toxicity was found, assessed by hematology and serum biochemistry. Absorbed doses in the tumour were high for both EGFR-targeted (30.4 Gy) and non-targeted AuNPs (21.9 Gy) but normal organ doses were very low (0.04–0.6 Gy) (Fig. 5d). In contrast to our previous in vitro study which showed an advantage of EGFR-targeting for increasing the absorbed dose and cytotoxicity of ^177^Lu-labeled radiation nanomedicines on MDA-MB-468 cells (Yook et al. 2015), in vivo in mice with s.c. MDA-MB-468 tumours, EGFR-targeting was not required. Instead, the most important observation was that after i.t. injection, AuNPs anchored ^177^Lu in the tumour, limiting redistribution to normal tissues, resulting in high absorbed doses in the tumour but very low doses in normal organs. This achieved tumour growth arrest but no observable normal tissue toxicity. In a subsequent study, we constructed HER2-targeted radiation nanomedicines by conjugating OPSS-PEG_5K_-trastuzumab and and OPSS-PEG_3K_-DOTA-[^177^Lu]Lu to AuNPs (30 nm) (Cai et al. 2017). These were specifically bound and internalized by HER2-positive BC cells in vitro and caused DNA DSBs that decreased the CS of these cells. Tumour growth in vivo was inhibited twofold after i.t. injection of 3 MBq (5.5 × 10^11^ AuNPs) of [^177^Lu]Lu-DOTA-PEG_3K_(trastuzumab-PEG_5K_)-AuNPs, but no normal tissue toxicity was found. In contrast to our earlier study that showed equivalent tumour growth inhibition for EGFR-targeted and non-targeted ^177^Lu-labeled AnNPs (Yook et al. 2016a), in this study, non-targeted [^177^Lu]Lu-DOTA-PEG_3K_-AuNPs were not as effective as [^177^Lu]Lu-DOTA-PEG_5K_-trastuzumab-AuNPs for inhibiting tumour growth and resulted in decreased hematocrit, red blood cell and platelet counts. Two factors might be contributing to the difference of the observation on therapeutic effects between targeted vs non-targeted: (1) the injected dose (3 MBq) in the HER2 targeted study was 50% lower than in the EGFR targeted study (4.5 MBq); (2) the tumour model (MDA-MB-361 xenografts in NOD/SCID mice, tumour doubling time of 5 d for untreated control) used in the HER2 targeted study was 3.5 times more aggressive than MDA-MB-468 xenografts in CD-1 athymic mice (tumour doubling time of 17 d for untreated control) used in the EGFR targeted study. It is worth noting that CD-1 athymic mice (which lack a thymus and T-cells) have more residual immune function than NOD/SCID mice (lack of functional T cells, B cells and natural killer cells). The residual immune function could enhance the therapeutic effect of ^177^Lu.

In these studies, we employed OPSS-PEG_5K_ polymers to attach [^177^Lu]Lu-DOTA and the monoclonal antibodies, panitumumab or trastuzumab to the surface of AuNPs, but the OPSS group forms a single gold-thiol bond with AuNPs that may not be stable in vivo, particularly in the presence of competing thiol-containing biomolecules [e.g. cysteine (Cys) or glutathione (GSH)]. Thus, we next compared the stability of AuNPs conjugated to three different metal-chelating polymers (MCPs) complexed to ^177^Lu: [^177^Lu]Lu-DOTA-PEG_4K_-OPSS, [^177^Lu]Lu-DOTA-PEG_4K_-lipoic acid (LA) and PEG_2K_-*p*Glu(DOTA-[^177^Lu]Lu)_8_-LA_4_. These MCPs form mono-thiol, di-thiol or multi-thiol bonds with AuNPs, respectively (Yook et al. 2016b). Dissociation of these polymers from AuNPs in the presence of the reducing agent, dithiothreitol (DTT) or Cys or GSH causes aggregation which results in a change in the spectroscopic properties of the AuNPs, which was used to assess the relative stability of the gold-thiol bonds. A multi-thiol linkage was the most stable in vitro to DTT, Cys or GSH challenge and to incubation in human plasma. AuNPs conjugated to PEG_2K_-*p*Glu(DOTA-[^177^Lu]Lu)_8_-LA_4_ resulted in the lowest liver uptake in vivo in non-tumour bearing athymic mice, but higher spleen uptake was noted compared to di-thiol MCPs. Thus, gold-thiol conjugation enabled by LA-functionalized polymers was used by our team to construct all subsequent radiation nanomedicines.

**Fig. 6:**
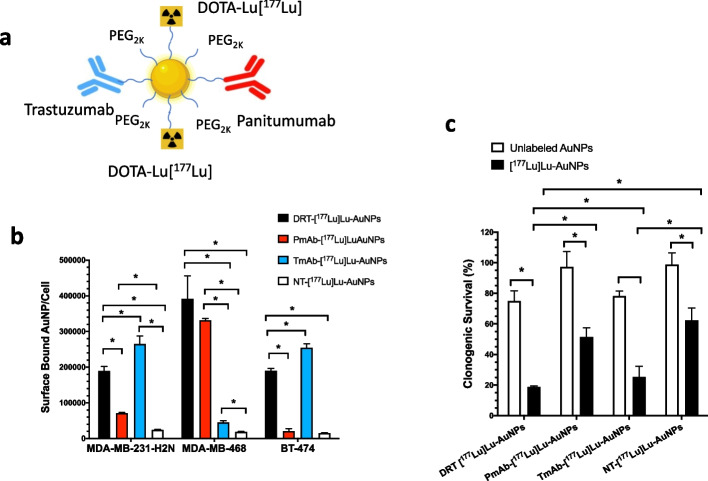
**a** Dual-receptor targeted (DRT) radiation nanomedicines composed of AuNPs modified with PEG linked to anti-HER2 trastuzumab (Tmab) or anti-EGFR panitumumab (Pmab) and to DOTA complexed with ^177^Lu. **b** Binding of DRT radiation nanomedicines or single-receptor targeted (SRT) PmAb-[^177^Lu]Lu-AuNPs or Tmab-[^177^Lu]Lu-AuNPs or non-targeted (NT) [^177^Lu]Lu-AuNPs to human breast cancer (BC) cells. MDA-MB-231-H2N cells express both HER2 and EGFR, MDA-MB-468 cells express only EGFR and BT-474 cells express high levels of HER2 but low EGFR. DRT radiation nanomedicines bound to BC cells expressing EGFR and/or HER2, while SRT radiation nanomedicines bound to cells that express EGFR or HER2. NT radiation nanomedicines exhibited low binding to BC cells. **c** Clonogenic survival of MDA-MB-231-H2N cells displaying both HER2 and EGFR treated with [^177^Lu]Lu-labeled DRT or SRT or NT radiation nanomedicines or unlabeled AuNPs. [^177^Lu]Lu-labeled DRT radiation nanomedicines were more cytotoxic than unlabeled AuNPs and were most potent for killing MDA-MB-231-H2N cells. * Significant differences (*P* < 0.05). Reprinted (adapted) with permission from: Yook, S. et al. Dual-receptor-targeted (DRT) radiation nanomedicine labeled with ^177^Lu is more potent for killing breast cancer cells that coexpress HER2 and EGFR than single-receptor-targeted (SRT) radiation nanomedicines. Mol. Pharm. 2020;17,1226–1236. Copyright 2020 American Chemical Society

We then constructed dual receptor-targeted (DRT) radiation nanomedicines that bind both HER2 and EGFR by conjugating trastuzumab-PEG_5K_-LA and panitumumab-PEG_5K_-LA and [^177^Lu]Lu-DOTA-PEG_3K_-LA to AuNPs (Yook et al. 2020) (Fig. 6a). These DRT radiation nanomedicines were intended to overcome receptor heterogeneity in BC tumours and exploit the co-expression of HER2 and EGFR by some trastuzumab-resistant BC cells (Gallardo et al. 2012). Single receptor-targeted (SRT) radiation nanomedicines were synthesized by conjugating only trastuzumab-PEG_5K_-LA or panitumumab-PEG_5K_-LA and [^177^Lu]Lu-DOTA-PEG_3K_-LA to AuNPs. DRT and SRT radiation nanomedicines were compared for binding and internalization into MDA-MB-231/H2N human BC cells that co-expressed moderate levels of HER2 and EGFR (HER2^mod^/EGFR^mod^), MDA-MB-468 cells cells with high levels of EGFR but negligible HER2 (EGFR^high^/HER2^neg^) or BT-474 cells with high HER2 but low EGFR (HER2^high^/EGFR^low^). Darkfield microscopy and cell binding assays (Fig. 6b) revealed that DRT radiation nanomedicines were bound by MDA-MB-231/H2N cells expressing both EGFR and HER2, while SRT forms were bound only by BC cells that displayed either EGFR or HER2. DRT were more effective in vitro than SRT radiation nanomedicines for decreasing the CS of MDA-MB-231/H2N cells that co-expressed HER2 and EGFR (Fig. 6c). Non-targeted radiation nanomedicines were less cytotoxic in vitro than either DRT or SRT forms. In the next section, we discuss the landscape of NPs labeled with β-particle emitting radionuclides for cancer treatment.

### Landscape: NPs labeled with β-particle emitting radionuclides

#### Lutetium-177

##### Gold nanoparticles (AuNPs)

Mendoza-Nava et al. (2017) synthesized dual-targeted ^177^Lu-labeled AuNPs that bound both the folate receptor-α (FR-α) and gastrin-releasing peptide receptor (GRPR). Poly(amidoamine) (G4 PAMAM) dendrimers were conjugated to folic acid and bombesin to target these receptors, respectively. The dendrimers were derivatized with p-SCN-benzyl-DOTA (Bn-DOTA) to complex ^177^Lu. These dendrimers were incorporated into the synthesis reaction of AuNPs. The resulting radiation nanomedicines were intended to enable combined hyperthermia and radiation treatment of BC. Hyperthermia is mediated by absorption of near infrared (nIR) light by AuNPs (Beik et al. 2016). Applying laser light at 532 nm (1.19 W/cm^2^) to T47D human BC cells that bound these AuNPs raised their temperature from 39.1 to 46.8 °C, and decreased their CS in vitro by > 85%. Moreover, [^177^Lu]Lu-Bn-DOTA-dendrimer-folic/acid-bombesin-AuNPs were fourfold more cytotoxic than non-targeted [^177^Lu]Lu-Bn-DOTA-dendrimer-AuNPs, decreasing the CS of T47D cells by > 90%, in agreement with the fourfold higher absorbed dose (63 Gy vs. 15 Gy, respectively).

Vilchis-Juarez et al. (2014) constructed AuNPs modified with cyclic arginine-glycine-aspartic acid (cRGD) peptides to target α_v_β_3_ integrins and conjugated these AuNPs to [^177^Lu]Lu-DOTA-GGC peptides. The size and charge of the [^177^Lu]Lu-DOTA-GGC-AuNPs-cRGD were 26 nm and − 64.6 mV, respectively. Intratumoural (i.t.) injection of 4 weekly amounts (2 MBq) of [^177^Lu]Lu-DOTA-GGC-AuNPs-cRGD in athymic mice with s.c. human C6 glioma xenografts decreased tumour volumes by 27-fold at 23 d compared to untreated mice. [^177^Lu]Lu-DOTA-GGC-AuNPs-cRGD targeting α_v_β_3_ integrins on C5 tumour cells were threefold more effective than non-targeted [^177^Lu]Lu-DOTA-GGC-AuNPs. Intratumoural (i.t.) injection resulted in high and retained ^177^Lu activity in the tumour. This deposited high absorbed doses in the tumour (64 Gy) but only low doses (< 1 Gy) in kidneys, liver and spleen. No kidney toxicity was detected. Interestingly, treatment of C6 tumours with [^177^Lu]Lu-DOTA-GGC-AuNPs-cRGD resulted in a decrease in the tumour uptake of [^18^F]F-2-fluorodeoxyglucose ([^18^F]FDG) compared to untreated mice, indicating a metabolic response to these radiation nanomedicines. In addition, tumour vascular endothelial growth factor (VEGF) gene expression was decreased, suggesting that [^177^Lu]Lu-DOTA-GGC-AuNPs-cRGD may have anti-angiogenic properties. The advantage of i.t. injection of these radiation nanomedicines is clearly shown by comparing these results to a previous report (Luna-Gutierrez et al. 2012) which found that the absorbed dose in s.c. U87MG human glioma tumours in mice after intraperitoneal (i.p.) administration of ^177^Lu-labeled AuNPs modified with RGD peptides to target α_v_β_3_ integrins was only 0.1–0.36 Gy, due to much lower tumour uptake, likely as a result of liver and spleen sequestration.

##### Micelles, polymers and dendrimers

PEGylated liposomes (100–116 nm) were synthesized by Petersen et al. (2016) that trapped DOTA for complexing ^64^Cu or ^177^Lu. This study focused on PET imaging with i.v. injected [^64^Cu]Cu-DOTA-liposomes to evaluate different levels of PEGylation (10 mol% vs. 5 mol%) on the tumour and normal tissue uptake in mice bearing s.c. H727 human neuroendocrine tumour xenografts. Tumour uptake ranged from 5 to 6% ID/g at 48 h p.i., and was maximized for 10 mol% PEGylation. Tumour doses were 0.11 Gy/MBq for small (2 g) tumours and 0.011 Gy/MBq for larger (20 g) tumours. These absorbed doses are very low compared to studies of ^177^Lu-labeled NPs administered by i.t. injection as discussed previously. Spleen and liver uptake were high (15–16% ID/g and 10–11% ID/g at 48 h p.i., respectively). The liver and spleen were among the normal organs receiving the highest radiation absorbed doses. Wang et al. (2020a) synthesized DOTA-tri-arginine-lipid micelles as a cationic cell-penetrating liquid form of brachytherapy labeled with ^177^Lu for treating tumours or with ^64^Cu for PET imaging. Retention in s.c. CT26 murine colon carcinoma tumours in Balb/c mice after i.t. injection of these [^64^Cu]Cu-DOTA-tri-arginine-lipid micelles was assessed. While i.t. injected [^64^Cu]CuCl_2_ or [^64^Cu]Cu-DOTA were rapidly eliminated from the tumour (< 10% ID/g remaining at 24 h), [^64^Cu]Cu-DOTA-tri-arginine-lipid micelles were cleared more slowly with 34% ID/g remaining at 6 h and 28% ID/g at 24 h p.i., suggesting the effects of cell-penetration mediated by the tri-arginine peptide.

Shi et al. (2021) synthesized semiconducting polymer nanoparticles (SPNs; ~ 207 nm) that absorb near infrared (nIR) light and enable photothermal therapy (PTT). These SPNs were modified with DOTA for complexing ^177^Lu and with glucose-dependent insulinotropic polypeptide (GIP) for targeting pancreatic cancer. Treatment of CFPAC human pancreatic cancer cells in vitro with these [^177^Lu]Lu-DOTA-GIP-SPNs (0.37–11.1 MBq/mL) combined with PTT by irradiation with a 808 nm laser (1 W cm^−2^, 5 min) reduced cell viability in a concentration-dependent manner to as low as 4.4%, while treatment with the highest concentration of [^177^Lu]Lu-DOTA-GIP-SPNs, only reduced cell viability to 34%. Non-radioactive DOTA-GIP-SPNs were not cytotoxic. In mice with s.c. CFPAC tumours, i.t. injection of [^177^Lu]Lu-DOTA-GIP-SPNs retained ^177^Lu activity in the tumour with no visible uptake in normal tissues by SPECT/CT. Treatment of mice with s.c. CFPAC tumours with 1.1 MBq of [^177^Lu]Lu-DOTA-GIP-SPNs injected i.t. combined with PTT arrested tumour growth, while [^177^Lu]Lu-DOTA-GIP-SPNs or PTT alone only slowed tumour growth compared to mice treated with non-radioactive DOTA-GIP-SPNs with or without PTT or normal saline-treated mice. These results are promising for local treatment of pancreatic cancer and suggest that combining PTT and ^177^Lu]Lu-DOTA-GIP-SPNs may be the most effective approach.

Hosseini et al. (2023) modified G4 PAMAM dendrimers with cetuximab for targeting EGFR and CHX-diethylenetriaminepentaacetic acid (CHX-DTPA) to complex ^177^Lu. G4 PAMAM dendrimers were relatively non-cytotoxic to EGFR-positive SW-480 human colon cancer cells in vitro, but conjugation of dendrimers to cetuxumab and especially, labeling with ^177^Lu increased cytotoxic potency. SPECT imaging in mice with s.c. SW-480 tumours showed tumour uptake at 24 h post i.v. injection. Tumour uptake was highest at 24 h p.i. (12% ID/g) but liver uptake was almost as high (11% ID/g). There was lower uptake in the spleen (2% ID/g) and kidneys (1% ID/g). No treatment or toxicity studies were performed.

##### Cellulose nanocrystals

Imlimthan et al. (2021) functionalized cellulose nanocrystals (CNCs) with DOTA for complexing ^177^Lu and poly-L-lysine to enable binding of the BRAF inhibitor, vemurafenib via electrostatic interaction to the CNCs. [^177^Lu]Lu-DOTA-verafenib-CNCs were cytotoxic in vitro to YUMM1.G1 and A375 melanoma cells in clonogenic assays. Biodistribution studies in mice with YUMM1.G1 tumours after i.v. injection of [^177^Lu]Lu-DOTA-verafenib-CNCs showed high but transient uptake in metastatic lungs reaching 47% ID/g at 6 h but decreasing to 18% ID/g at 72 h p.i. Liver and spleen uptake were high (18–26% ID/g and 28–60% ID/g, respectively). Treatment of mice with YUMM1.G1 lung metastases with two amounts (2 MBq) of [^177^Lu]Lu-DOTA-verafenib-CNCs separated by 10 days improved median survival to 27 d vs. 17 d for [^177^Lu]Lu-DOTA-CNCs not bound to vemurafenib and 13 d for vemurafenib alone or 12 d for vehicle-treated mice. However, fatal secondary metastases formed in the cardiac muscle and thoracic cavity that could not be effectively treated, which would prevent further development of these NPs.

##### Inorganic NPs

Salvanou et al. (2022) constructed calcium alginate and PEGylated magnetic iron oxide NPs (MIONPs; ~ 120 nm) and labeled these to high efficiency (90–95%) by direct binding of ^177^Lu or ^68^Ga in Na acetate buffer, pH 5.5 or 4.0, respectively, heated at 75 °C for 30 min. ^177^Lu or ^68^Ga-labeled MIONPs were relatively stable in serum (> 70% up 7 d at 37 °C). Non-radioactive MIONPs were not cytotoxic to 4T1 murine mammary carcinoma cells in vitro, but ^177^Lu-labeled MIONPs (4 MBq/mL) modestly decreased cell proliferation. Biodistribution was studied only in healthy mice but there was high uptake in the liver (20–27% ID/g) and spleen (10–13% ID/g) after i.v. injection. No in vivo treatment studies were conducted. Stankovic et al. (2023) constructed superparamagnetic iron oxide NPs (SPIONs; ~ 11 nm) coated with dimercaptosuccinic acid (DMSA) which were labeled to high efficiency (86%) with ^177^Lu. These [^177^Lu]Lu-DMSA-SPIONs were stable in vitro in serum up to 6 d at 37 °C. Following i.t. injection in Balb/c mice with s.c. CT26 or 4T1 tumours, uptake in these tumours was high and strongly retained (> 95%) up to 11 d p.i. Similar to other studies that used i.t. injection of radiolabeled NPs, there was minimal uptake (< 1% ID/organ) in the liver, kidneys, spleen or lungs. However, in mice with 4T1 tumours, liver uptake increased to 19.8% ID/organ, then subsequently decreased to 4.3% ID/organ at 14 d.i. Administration of a single amount (3.7 MBq) of [^177^Lu]Lu-DMSA-SPIONs in mice with 4T1 or CT26 tumours slowed tumour growth compared to mice treated with non-radioactive DMSA-SPIONs or untreated mice, but did not arrest the growth or decrease the size of these tumours. A second administration after 5 d did not improve tumour response. Similar tumour growth inhibition was observed for a range of administered activity (1.85–9.25 MBq) of [^177^Lu]Lu-DMSA-SPIONs. Histopathological staining showed slight toxicity to the liver and kidneys.

Zhang et al. (2020) synthesized PEGylated silica NPs (~ 6 nm) modified with α-melanocyte stimulating hormone (α-MSH) peptides to target melanocortin-1 receptors on melanoma cells. These NPs were conjugated to DOTA and efficiently (> 95%) complexed ^177^Lu. [^177^Lu]Lu-DOTA-α-MSH-NPs were stable (> 90%) in vitro in serum up to 24 h at 37 °C. [^177^Lu]Lu-DOTA-α-MSH-NPs were bound and internalized in vitro by B16F10 and M21 melanoma cells. In SCID mice with s.c. B16F10 tumours or C57BL6 mice with M21 tumours, tumour uptake at 24 h post i.v. injection was 9.6% ID/g and 9.3% ID/g, respectively. In SCID mice, liver and spleen uptake were low (5.8% ID/g and 5.1% ID/g, respectively) but in C57BL6 mice, uptake in these organs was 1.5-fold higher. [^177^Lu]Lu-DOTA-α-MSH-NPs were eliminated mainly by renal excretion with almost 80% excreted in the urine by 96 h p.i. Dosimetry showed that the kidneys received the highest absorbed doses (3.8 cGy/37 MBq) but bone marrow and intestine doses were low (0.12 cGy/37 MBq). The MTD in mice was 37 MBq based on loss of body weight. Treatment of mice with s.c. M21 tumours with 18.5 or 37 MBq of [^177^Lu]Lu-DOTA-α-MSH-NPs administered i.v. increased median survival to 58 and 43 d, respectively, compared to mice treated with non-radiolabeled NPs (41 d) or no treatment (27 d). Administration of 18.5 or 37 MBq of non-targeted [^177^Lu]Lu-DOTA-NPs increased median survival to 33 d or 60 d, respectively. In this case, tumour uptake was most likely mediated by the EPR effect. Treatment of mice with B16F10 tumours with 18.5 MBq of [^177^Lu]Lu-DOTA-α-MSH-NPs increased the median survival to 25 d versus 21 d for non-targeted [^177^Lu]Lu-DOTA-NPs, 14 d for non-radioactive NPs, or 16 d for untreated mice.

##### Neutron-activated lutetium oxide NPs

An interesting approach to construction of radiation nanomedicines was reported by Ancira-Cortez et al. (2020). Stable lutetium oxide (^Nat^Lu_2_O_3_) NPs (~ 27 nm) were synthesized, then neutron-activated in a reactor (1 × 10^13^ n sec^−1^ cm^−2^ for 20 h) to produce [^177^Lu]Lu_2_O_3_ NPs. These NPs were then modified with prostate-specific membrane antigen (PSMA)-binding peptides by reaction with DOTA-HYNIC-iPSMA through the DOTA moiety which complexes lutetium. The [^177^Lu]Lu_2_O_3_-DOTA-HYNIC-iPSMA NPs were specifically bound and internalized in vitro by PSMA-positive HepG2 human hepatocellular carcinoma cells, and reduced their viability to 53% versus 69% for non-targeted [^177^Lu]Lu_2_O_3_ NPs. The cytotoxicity of non-targeted NPs was explained by non-specific uptake by HepG2 cells combined with the cross-fire effect of the 2 mm range β-particles emitted by ^177^Lu. At 24 h post i.v. injection in healthy Balb/c mice, the [^177^Lu]Lu_2_O_3_-DOTA-HYNIC-iPSMA NPs localized in the liver (9% ID/g) with lower uptake in the spleen (0.8% ID/g) and kidneys (0.7% ID/g). Interesting images were obtained showing the liver uptake of these [^177^Lu]Lu_2_O_3_-DOTA-HYNIC-iPSMA NPs, by taking advantage of the luminescence of ^Nat^Lu_2_O_3_ when exposed to the β-particle and γ-photon emissions of ^177^Lu. In a subsequent report (Luna-Gutierrez et al. 2022), stable ^Nat^Lu_2_O_3_ NPs were neutron-activated to [^177^Lu]Lu_2_O_3_ NPs in a nuclear reactor, then modified with DOTA-HYNIC-iPSMA or an inhibitor of fibroblast activation protein ligand (DOTA-HYNIC-iFAP). Intratumoural (i.t.) injection of these [^177^Lu]Lu_2_O_3_-DOTA-HYNIC-iPSMA or [^177^Lu]Lu_2_O_3_-DOTA-HYNIC-iFAP NPs in mice with s.c. HCT116 human colon cancer xenografts resulted in very high tumor uptake that was retained up to 96 h p.i. (77–84% ID/g) with minimal uptake in the liver (~ 1% ID/g) or spleen (< 0.5% ID/g). Mice treated with 1–2 MBq of [^177^Lu]Lu_2_O_3_-DOTA-HYNIC-iPSMA or [^177^Lu]Lu_2_O_3_-DOTA-HYNIC-iFAP NPs showed strong tumor growth inhibition compared to untreated mice. Non-targeted [^177^Lu]Lu_2_O_3_ NPs also inhibited tumor growth but were less effective than the targeted NPs. There was no liver or kidney toxicity observed. The absorbed doses in the tumour were very high (> 100 Gy). These results are very promising for application of [^177^Lu]Lu_2_O_3_-DOTA-HYNIC-iPSMA or [^177^Lu]Lu_2_O_3_-DOTA-HYNIC-iFAP NPs for local treatment of tumours expressing PSMA or FAP. Particularly fascinating was that this report included a first-in-human study of a patient with widespread unresectable liver metastases from colon cancer who was administered 185 MBq of [^177^Lu]Lu_2_O_3_-DOTA-HYNIC-iPSMA NPs by i.v. injection. SPECT/CT imaging demonstrated uptake of [^177^Lu]Lu_2_O_3_-DOTA-HYNIC-iPSMA NPs in liver metastases, which were further identified as PSMA-positive by SPECT imaging with [^99m^Tc]Tc-iPSMA. Although in this human study the radiation nanomedicine was administered i.v., based on dosimetry calculations, it was estimated that up to 1,350 MBq could be safely administered to humans without exceeding the tolerable doses in normal organs, and this administered amount may deposit 42–202 Gy in liver metastases.

#### Rhenium-186

Rhenium-186 [^186^Re; half-life (t_1/2_) = 3.8 d] decays by β-particle emission (E_βmax_ = 1.09 MeV; 92.5%) to stable osmium-186 (^186^Os) or by electron capture (EC) to tungsten-186 (^186^W; 7.5%) with emission of a γ-photon (Eγ = 136 keV) that allows SPECT imaging. Soundararajan et al. (Soundararajan et al. 2011) complexed ^186^Re to N,N-bis(2-mercaptoethyl)-N',N'-diethylethylenediamine (BMEDA) chelators that were incorporated into Dox-containing liposomes. They also synthesized ^186^Re-labeled PEGylated liposomes without Dox. The MTD in nude rats after i.v. administration was 740 MBq/kg for [^186^Re]Re-BMEDA-Dox liposomes and 1,480 MBq/kg for [^186^Re]Re-BMEDA-PEG-liposomes, due to dose-limiting hematopoietic toxicity, which recovered at 28 d p.i. Additionally, the [^186^Re]Re-BMEDA-Dox liposomes caused gastrointestinal toxicity. Treatment of nude rats with s.c. head and neck squamous cell carcinoma (SCC-4) tumours at the MTD slowed tumour growth. [^186^Re]Re-BMEDA-Dox liposomes were more effective than [^186^Re]Re-BMEDA-PEG-liposomes. At the MTD, the absorbed doses in the tumour for [^186^Re]Re-BMEDA-Dox liposomes and [^186^Re]Re-BMEDA-PEG-liposomes were 46 Gy and 32 Gy, respectively. Normal organ doses were 68 Gy, 38 Gy and 288 Gy for the lungs, liver and kidneys, respectively in rats injected with [^186^Re]Re-BMEDA-Dox liposomes and 57 Gy, 71 Gy and 425 Gy for rats receiving [^186^Re]Re-BMEDA-PEG-liposomes. Thus, although these results suggest that a combined chemoradiation therapy approach may be promising for treating tumours, there were high normal organ absorbed doses and normal tissue toxicity for these i.v. administered radiation nanomedicines. To improve their safety and dosimetry, Hyrcushko et al. (2011) instead intratumorally (i.t.) infused [^186^Re]Re-BMEDA-PEG-liposomes in nude rats with s.c. SCC-4 tumours and estimated the absorbed doses in tumours for correlation with response to treatment. A single i.t. infusion (185 MBq/cm^3^ of tumour) or three fractionated i.t. infusions to more evenly space activity distribution in the tumour, as well as i.p. injection of anti-vascular endothelial growth factor (VEGF) bevacizumab (Avastin^®^; Genentech) prior to infusion of the [^186^Re]Re-BMEDA-PEG-liposomes were studied. Multiple fractionated infusions improved tumour dose homogeneity and bevacizumab improved retention of [^186^Re]Re-BMEDA-liposomes in the tumour by disrupting tumour vascularization. No correlation was found between overall tumour dose and response, but the Equivalent Uniform Dose (EUD) which assessed the tumour dose heterogeneity predicted tumour response. In a follow-up study, French et al. (2010) examined the effectiveness of [^186^Re]Re-BMEDA-PEG-liposomes infused i.t. in three fractionated amounts (total = 185 MBq/cm^3^ of tumour) for treatment of s.c. SCC-4 tumours in nude rats. Tumour growth was strongly inhibited for rats infused i.t. with [^186^Re]Re-BMEDA-PEG-liposomes, while rats receiving unlabeled liposomes or non-liposomal [^186^Re]Re-perrhenate or [^186^Re]Re-BMEDA showed rapid tumour growth. In rats infused with [^186^Re]Re-BMEDA-PEG-liposomes, tumour activity was 48% ID/g after i.t. infusion but this decreased to 9% ID/g at 140 h p.i. However, non-liposomal [^186^Re]Re-sodium perrhenate and [^186^Re]Re-BMEDA were rapidly eliminated from the tumour in 2–4 h. Absorbed doses in the tumour were high (526 Gy). [^186^Re]Re-BMEDA-PEG-liposomes were not toxic to normal tissues, causing only a minor decrease in body weight and no evidence of hematopoietic toxicity. This study further highlights the major advantages of i.t. administration of radiation nanomedicines for improving their effectiveness for treating tumours and ability to minimize normal organ doses and toxicity.

#### Rhenium-188

^188^Re (t_1/2_ = 16.9 h) decays to ^188^Os by β-particle emission (Eβmax = 2.12 MeV; 85%) and emits a γ-photon [Eγ = 155 keV (15%)] that allows SPECT imaging. Liu et al. (2012) synthesized [^188^Re]Re-BMEDA-PEG-liposomes and compared their effectiveness after i.v. injection to liposomal Dox (Lipo-Dox) for treating 4T1 murine mammary carcinoma tumours in Balb/c mice. SPECT/CT and biodistribution studies were used to assess tumour and normal tissue uptake of [^188^Re]Re-BMEDA-PEG-liposomes. Maximum tumour uptake occurred at 24 h p.i. (3.0% ID/g) but there was high liver (4.6% ID/g) and spleen (3.0% ID/g) uptake at this time point, and excretion in the feces (4.7% ID/g). The MTD for [^188^Re]Re-BMEDA-PEG-liposomes was 37 MBq and for Lipo-Dox was 25 mg/kg, but the MTD was identified based on body weight loss or treatment-induced death, and did not assess blood cell counts or serum biochemistry. SPECT/CT showed tumour uptake and uptake in the liver and spleen. Lipo-Dox (20 mg/kg) was most effective for decreasing the growth of small (~ 50 mm^3^) and large (~ 300 mm^3^) 4T1 tumours and for prolonging survival. [^188^Re]Re-BMEDA-PEG-liposomes (29.6 MBq) slowed tumour growth compared to untreated mice and slightly to modestly improved survival.

[^188^Re]Re-BMEDA-PEG-DXR-liposomes, where DXR is doxorubicin, were studied for treatment of s.c. CT26 tumours in Balb/c mice after i.v. injection of three amounts (22.2 MBq) separated by 4 d (Chang et al. 2010). Peak tumour uptake occurred at 24 h p.i. (6.1% ID/g). At this time point, spleen uptake was high (20.2% ID/g) but liver uptake (5.9% ID/g) and kidney uptake (3.3% ID/g) were lower. Tumour size decreased by treatment with [^188^Re]Re-BMEDA-PEG-DXR-liposomes and tumours were growth-arrested by [^188^Re]Re-BMEDA-PEG-liposomes without Dox. Tumour growth was inhibited by Lipo-Dox compared to normal saline treated mice, but this was less effective than the ^188^Re-labeled liposomes. [^188^Re]Re-BMEDA-PEG-DXR-liposomes were the most effective for prolonging survival, followed by [^188^Re]Re-BMEDA-PEG-liposomes while Lipo-Dox did not significantly improve survival compared to normal saline-treated mice. Tsai et al. (2011) studied the biodistribution and pharmacokinetics of [^188^Re]Re-BMEDA-PEG-liposomes and compared their effectiveness to 5-fluorouracil (5-FU) for treating C26 murine colon carcinoma peritoneal metatastases after i.v. injection in Balb/c mice. Blood activity decreased from 41 to 1.4% ID/g over 72 h. Uptake in metastases was maximal at 16–24 h p.i. (7.4–7.9% ID/g). The highest normal tissue uptake at these time points was in the liver (5.2–6.6% ID/g), spleen (7.5–7.6% ID/g) and kidneys (4.2–5.0% ID/g). SPECT imaging showed localization of [^188^Re]Re-BMEDA-PEG-liposomes in metastases in the peritoneal cavity. Based on body weight loss, the MTD for [^188^Re]Re-BMEDA-PEG-liposomes was 37 MBq. Encouragingly, a single administration of [^188^Re]Re-BMEDA-PEG-liposomes at 80% of the MTD (29.6 MBq) prolonged survival of mice with peritoneal metastases (32.8 d) compared to mice treated with 5-FU (26.7 d) or receiving no treatment (24.3 d). Chen et al. (2008) studied local intraperitonal (i.p.) injection of [^188^Re]Re-BMEDA-PEG-DXR-liposomes co-loaded with Dox for treatment of peritoneal C26 tumours in Balb/c mice. These tumours form ascites. ^188^Re activity in the ascites decreased from 49 to 3.9% ID/g at 96 h p.i. Tumour uptake was maximal at 48 h p.i. (1.9% ID/g). Spleen uptake at 48 h p.i. was very high (31.2% ID/g) but liver and kidney uptake were lower (5.2% ID/g and 2.0% ID/g, respectively). The MTD of [^188^Re]Re-BMEDA-PEG-DXR-liposomes based on body weight loss and a transient decrease in WBC and platelet counts at 1–2 weeks post-treatment was 29.6 MBq. Treatment of tumour-bearing mice with 22.2 MBq of [^188^Re]Re-BMEDA-PEG-DXR-liposomes was more effective than [^188^Re]Re-BMEDA-PEG-liposomes or Lipo-Dox for prolonging survival (29.3 d vs. 23.2 d and 22.2 d, respectively). In another study, lung metastases from CT26 colon cancer tumours in Balb/c mice were treated by i.v. injection of 29.6 MBq of [^188^Re]Re-BMEDA-PEG-liposomes (Chen et al. 2012). The median survival of these mice increased to 58 d versus 42–43 d for mice treated with unlabeled liposomes or normal saline. Treatment with 5-FU was less effective (median survival = 49 d). Tumour uptake reached 5.5% ID/g at 24 h and uptake in the spleen, liver and kidneys at this time point were 6.9% ID/g, 8.2% ID/g and 4.4% ID/g, respectively. The advantage of local i.p. vs. i.v. administration of [^188^Re]Re-BMEDA-PEG-liposomes was further demonstrated in mice with intraperitoneal ES-2 human ovarian cancer tumours (Shen et al. 2016). These tumours were radiation-resistant and expressed cancer stem cell (CSC) biomarkers (CD44^high^/CD24^high^/CD133^high^). Treatment with [^188^Re]Re-BMEDA-PEG-liposomes at one third the MTD administered i.v. was not effective, but i.p. administration controlled tumour growth in the peritoneum assessed by bioluminescence imaging (BLI) and prolonged survival compared to untreated mice. Moreover, i.p. [^188^Re]Re-BMEDA-PEG-liposomes reversed the glycolytic stem cell phenotype of these cells back to oxidative phosphorylation and decreased expression of CSC biomarkers on tumour cells.

#### Yttrium-90

^90^Y (t_1/2_ = 64 h) decays to ^90^Zr by β-particle emission (E_βmax_ = 2.2 MeV; 100%) but does not emit a γ-photon, thus imaging is limited to detecting the Bremsstrahlung radiation resulting from interaction of β-radiation with tissues, which has poor spatial resolution. Radovic et al. (2015) constructed spherical iron oxide (Fe_2_O_3_) nanoparticles (IONPs) that were not modified with PEG (naked; 80 nm) or were PEGylated (46 nm) and further incorporated ^90^Y. The aim was to synthesize multi-functional IONPs that may be used for combined magnetic hyperthermia and radiotherapy. After i.v. injection in rats, there was high liver uptake of naked and PEGylated [^90^Y]Y-IONPs, but the lung uptake was sixfold higher for naked [^90^Y]Y-IONPs. No studies were performed to examine their therapeutic potential. Ognjanovic et al. (2019) synthesized IONPs with a “flowerlike” structure and labeled these with ^90^Y, ^177^Lu or ^99m^Tc to a high efficiency (> 97%). Magnetic hyperthermia with unlabeled IONPs killed mouse CT-26 colon cancer cells, but without magnetic hyperthermia there was no cytotoxicity. The effects of the radiation emitted by ^90^Y, ^177^Lu or ^99m^Tc on CT-26 cells were not studied. However, in a subsequent report, Vukadinovic et al. (2022) evaluated the tumour growth inhibitory effects of these [^90^Y]Y-IONPs after i.t. injection in mice with s.c. CT-26 or 4T1 murine mammary carcinoma tumours combined with magnetic hyperthermia. Intratumoral (i.t.) injection of 1.85 MBq [^90^Y]Y-IONPs alone or combined with magnetic hyperthermia inhibited tumour growth. In contrast, i.v. administration of these [^90^Y]Y-IONPs resulted in low tumour uptake that was not considered feasible for therapy studies. Guryev et al. (2018) constructed NPs (~ 50 nm) composed of a sodium yttrium fluoride (NaYF_4_) core that incorporated Na[^90^Y]YF_4_ surrounded by a shell of ytterbium (Yb), thulium (Tm) and NaYF_4_. These NPs were then conjugated to *Pseudomonas* Exotoxin (PE40) fused to a designed ankyrin repeat protein (DARPin) that binds HER2. These targeted Na[^90^Y]YF4 NPs were cytotoxic in vitro to HER2-positive SK-BR-3 human BC cells and inhibited the growth of SK-BR-3 tumour xenografts in vivo in athymic mice. Our team has studied ^90^Y-labeled AuNPs incorporated into a nanoparticle depot (nanodepot; NPD) for local treatment of 4T1 murine mammary carcinoma tumours in mice (see subsequent Scientific Journey section: *Nanodepot for i.t. delivery of *^*90*^*Y-labeled NPs*).

#### Gold-198

^198^Au decays (t_1/2_ = 2.7 d) decays to stable ^198^Hg by β-particle emission (Eβmax = 0.96 MeV; 99%) and emits a γ-photon [Eγ = 411 keV (96%)] that may be imaged by SPECT. ^198^Au is attractive for synthesizing radiation nanomedicines because it may be incorporated directly into AuNPs and does not require conjugating metal-chelating polymers for radiolabeling. This makes ^198^AuNPs easy to synthesize and stable, since they are more resistant to loss of radiometal. Nonetheless, taking a different synthetic approach, Xuan et al. (2020) produced ^198^Au gold nanoclusters (^198^AuNCs) by neutron irradiation of stable [^197^Au]AuNCs in a nuclear reactor using the ^197^Au(n, γ)^198^Au reaction. The cytotoxicity of these ^198^AuNCs was assessed in vitro on human PC-3 pancreatic cancer cells, MDA-MB-231 human breast cancer (BC) cells and MV3 melanoma cells. At absorbed doses of 2 Gy and 4 Gy, ^198^AuNCs were as cytotoxic as 100 nM paclitaxel against all three cell lines. No in vivo therapeutic studies were performed. Black et al. (2014) synthesized different shapes (spheres, disks, rods and cages) of PEGylated AuNPs incorporating ^198^Au that each had a similar size (~ 50 nm) and studied their tumour and normal tissue uptake after i.v. injection in mice with s.c. EMT6 murine mammary carcinoma tumours. Spherical ^198^AuNPs exhibited the longest residence time in the blood, while other shapes of ^198^AuNPs were cleared quickly from the blood. Spleen uptake of spherical ^198^AuNPs was only ~ 6% ID/g at 24 h p.i., but was much greater (50–100% ID/g) for other shapes. Liver uptake was high for spherical ^198^AuNPs (~ 50% ID/g) but was even higher for disk, rod or cage-shaped ^198^AuNPs. Interestingly, Cerenkov radiation caused by interaction of the β-particles emitted by ^198^Au with tissues was used to image the tumour and normal organ uptake of ^198^AuNPs. Unusually high tumour uptake for i.v. administration was found for spherical ^198^AuNPs (23.2% ID/g at 24 h p.i.), but this was lower for disks (4.9%ID/g), rods (< 2.0% ID/g) or cages (7.5% ID/g). The results of this study suggest that spherical AuNPs may be best suited to construct radiation nanomedicines.

#### Other β-particle emitting radionuclides

Kao et al. (2013) conjugated PEGylated AuNPs (53 nm) to ^123^I/^131^I-labeled anti-EGFR C225 mAbs. ^131^I (t_1/2_ = 8 d) decays to ^131^Xe, emitting β-particles [E_βmax_ = 0.60 MeV (89.9%) and 0.33 MeV (7.3%)] and an imageable γ-photon [Eγ = 364 keV (82%)]. ^123^I (t_1/2_ = 13.2 h) is an AE-emitter that decays by EC to ^123^Te emitting a γ-photon [Eγ = 159 keV (83.2%)] for SPECT imaging. Confocal fluorescence microscopy revealed that C225-PEG-AuNPs were bound and internalized into EGFR-positive A431 human lung cancer cells in vitro. Using a cell-proliferation assay, it was found that [^131^I]I-C225-PEG-AuNPs reduced the viability of A431 cells to ~ 37% versus 82% for treatment with free ^131^I. A431 tumors were imaged by SPECT in mice up to 4 h post-i.v. injection, but tumour activity was cleared by 8 h p.i. There was high liver and spleen uptake.

### Journey: nanoparticle depot for i.t. delivery of ^90^Y-labeled NPs

**Fig. 7:**
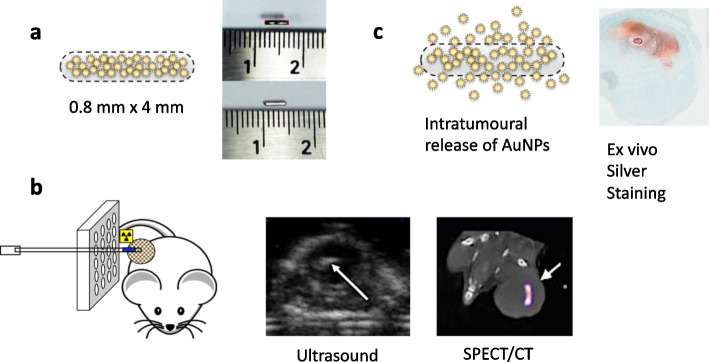
**a** Nanoparticle Depot (NPD) composed of calcium alginate and incorporating AuNPs. The size of the NPD (top panel) is compared to a conventional brachytherapy seed (bottom panel). **b** The NPD may be inserted into a tumour in a mouse using a brachytherapy seeding needle guided by a template. Once inserted, the NPD may be visualized by ultrasound imaging or by SPECT/CT when loaded with AuNPs labeled with ^111^In. **c** The NPD is porous allowing release of small AuNPs into a tumour (left panel) to aid in homogenizing the absorbed dose from the radiation nanomedicine. Release of small (5 nm) AuNPs from a NPD into a MDA-MB-231 human breast cancer (BC) tumour xenograft in a mouse were detected ex vivo by silver-enhanced staining. Reprinted (adapted) with permission from: Lai, P. et al. Depot system for controlled release of gold nanoparticles with precise intratumoral placement by permanent brachytherapy seed implantation (PSI) techniques. Int. J. Pharm. 2016;515:729–739

Although i.t. injection of radiation nanomedicines offers major advantages in maximizing the uptake and retention of activity in tumours and minimizing normal tissue uptake yielding high absorbed doses in tumours with low doses in normal organs, direct i.t. injection results in unpredictable and heterogeneous distribution of activity and consequently heterogeneous dose distribution. For example, we found that i.t. injection of 4.5 MBq of EGFR-targeted [^177^Lu]Lu-DOTA-PEG_4K_(panitumumabPEG_5K_)-AuNPs or non-targeted [^177^Lu]Lu-DOTA-PEG_4K_-AuNPs into s.c. MDA-MB-468 tumours in mice resulted in wide radioactivity distribution ranging from 0 to 2750 Bq and 0–400 Bq, respectively (Yook et al. 2016a). Although the maximum 2 mm penetration of the β-particles emitted by ^177^Lu mitigates the heterogeneity in dose distribution by a “cross-fire” effect, the absorbed doses distribution were also wide and ranged from 0–1300 Gy for [^177^Lu]Lu-DOTA-PEG_4K_(panitumumab-PEG_5K_)-AuNPs and 0–250 Gy for [^177^Lu]Lu-DOTA-PEG_4K_-AuNPs. For clinical application of radiation nanomedicines, it is important to have predictable, homogeneous, and sufficiently high absorbed doses in the tumour to assure that a minimum dose is received in all regions to cause several logarithms of tumour cell killing. Thus, in the next step in our journey, we constructed a nanoparticle depot (NPD) that incorporated radiolabeled AuNPs and could be precisely inserted into tumours utilizing clinically-used brachytherapy seed needle insertion techniques to achieve predictable dose delivery (Lai et al. 2016) (Fig. 7a) The NPD dimensions (0.8 mm × 4 mm) were comparable to a conventional brachytherapy seed. The NPD was composed of cross-linked 6, 8 or 10% calcium alginate, a biocompatible material (Hurtado et al. 2022). We loaded the NPD with 9.5 × 10^13^ 5 nm AuNPs, 3.5 × 10^12^ 15 nm AuNPs, 4.4 × 10^11^ 30 nm AuNPs or 9.5 × 10^10^ 50 nm AuNPs. The NPD inserted into a tumour may be visualized by ultrasound imaging or by SPECT/CT when the incorporated AuNPs are labeled with ^111^In Fig. 7b. The NPD was porous which permitted release of smaller sized AuNPs into a tumour to aid in homogenizing the dose distribution. The amount of AuNP release in a tissue-equivalent phantom was dependent on AuNP size with 100%, 98%, 7% or 3.3% released over a 3 d period for 5, 15, 30 or 50 nm AuNPs, respectively. Release was not dependent on the percentage of calcium alginate used to construct the NPD. Release of small (5 nm) AuNPs from the NPD inserted into a s.c. MDA-MB-231 human BC tumour in a SCID mouse was visualized ex vivo by silver enhanced staining (Fig. 7c).

**Fig. 8:**
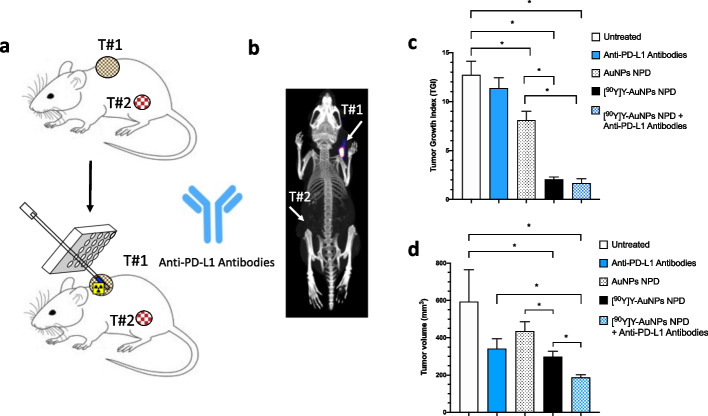
**a** Experimental design for treatment of s.c. 4T1 murine mammary carcinoma tumours in immunocompetent Balb/c mice. [^90^Y]Y-AuNPs (3.5 MBq; 1.0 × 10^14^ AuNPs) or unlabeled AuNPs were incorporated into a NPD and inserted into one tumour (T#1) in a mouse with two tumours. The second tumour (T#2) was located outside the range of the β-particles emitted by ^90^Y (maximum range = 12 mm). Some mice also received intraperitoneal (i.p.) injection of anti-PD-L1 antibodies (10 mg/kg 3 times per week). Control mice received anti-PD-L1 antibodies alone or a NPD incorporating unlabeled AuNPs or were untreated. **b** SPECT/CT image of a mouse inserted i.t. with a NPD loaded with [^111^In]In-AuNPs revealed retention of activity in T#1. **c** Insertion of the NPD loaded with [^90^Y]Y-AuNPs into T#1 strongly inhibited tumour growth at 14 d post-treatment with/without co-administration of anti-PD-L1 antibodies. **d** An abscopal growth-inhibitory effect was observed for T#2 by treating T#1 with a NPD incorporating [^90^Y]Y-AuNPs. This effect was enhanced by co-administration of anti-PD-L1 antibodies. *Significant differences (*P* < 0.05). Adapted and reprinted with permission from: Cai, Z. et al. ^90^Y-labeled gold nanoparticle depot (NPD) combined with anti-PD-L1 antibodies strongly inhibits the growth of 4T1 tumors in immunocompetent mice and induces an abscopal effect on a distant non-irradiated tumor. Mol. Pharm. 2022;19:4199–4211. Copyright 2022 American Chemical Society

We subsequently studied a NPD incorporating 3.5 MBq of PEG_2K_-*p*Glu(DOTA-[^90^Y]Y)_8_-LA_4_-AuNPs; (1 × 10^14^ AuNPs; 12 nm) inserted i.t. in a s.c. 4T1 murine mammary carcinoma tumour in immunocompetent Balb/c mice that had two 4T1 tumours (Cai et al. 2022) (Fig. 8a). One of these tumours (primary tumour) was treated with a radiation nanomedicine-containing NPD while the other (secondary tumour) remained untreated. The secondary tumour was located outside the maximum 12 mm range of the β-particles emitted by ^90^Y, thus was not irradiated by the radiation nanomedicine deposited in the primary tumour. In this study, we were interested to investigate if treatment of the primary tumour with the NPD incorporating PEG_2K_-*p*Glu(DOTA-[^90^Y]Y)_8_-LA_4_-AuNPs would cause an immune-mediated abscopal effect on the distant tumour, particularly with co-administration of anti-Programmed Cell Death Ligand-1 (anti-PD-L1) checkpoint antibodies. The abscopal (“out-of-scope”) effect was first described by Mole in 1953 who observed that X-irradiation of one tumour in a mouse with two tumours inhibited the growth of the second tumour (Mole 1953). The mechanism is believed to be due to release of damage-associated molecular patterns (DAMPs) by killed tumour cells that stimulate the immune system resulting in a cytotoxic T-cell response that inhibits the growth of the non-irradiated second tumour (Hu et al. 2017). There have been some reports of an abscopal effect in cancer patients treated with radiation, but it is rare due to immune checkpoints that block cytotoxic T-cell responses (Abuodeh et al. 2016). However, the introduction of checkpoint immunotherapy may increase the likelihood of an abscopal effect (Reynders et al. 2015) and there is a growing interest in combining radiation with immune checkpoint blockade (Nguyen et al. 2021). Imaging (Fig. 8b) and biodistribution studies after insertion of the NPD demonstrated that ^90^Y activity was retained in the primary (treated) tumour with > 100–400 fold more ^90^Y in this tumour than in normal tissues [400–800% injected dose/g (%ID/g) vs. < 0.5% ID/g] except for the kidneys (< 5% ID/g). The uptake of ^90^Y in the distant tumour was very low (< 0.2% ID/g). Consequently, the radiation absorbed dose in the primary tumour was very high (472 Gy), while the dose in the secondary tumour was very low (0.13 Gy). Normal organ doses (0.04–0.8 Gy) were also low, except for the kidneys (4 Gy). Radiation nanomedicine treatment of the primary tumour arrested the growth of this tumour, but in addition, inhibited the growth of the distant secondary tumour by an abscopal effect (Fig. 8c). This effect was enhanced by administering anti-mouse PD-L1 antibodies. However, anti-PD-L1 antibodies alone or a NPD incorporating non-radioactive AuNPs only slightly inhibited tumour growth. There was no normal tissue toxicity. Body weight was unchanged indicating no general toxicity and there was no increase in blood alanine aminotransferase (ALT) or creatinine (Cr) indicating no liver or kidney toxicity, respectively. Blood cell counts were normal, except for white blood cells (WBCs) which were decreased. However, 4T1 tumours secrete cytokines (e.g. G-CSF) that increase WBC counts (DuPre and Hunter 2007). Healthy non-tumour bearing Balb/c mice had much lower WBC counts than tumour-bearing mice. Thus, we interpreted the decreased WBC counts in mice treated with the radiation nanomedicine vs. untreated tumour-bearing mice as response to treatment (i.e. lower tumour burden) rather than hematopoietic toxicity. These results are very encouraging for combining i.t. delivered radiation nanomedicines with checkpoint immunotherapy since they raise the intriguing idea that this approach may extend radiation nanomedicines from a local treatment to one that is both a local and a systemic treatment by harnessing a cytotoxic T-cell immune response.

We next modeled the dose distribution rate from a NPD inserted into a s.c. MDA-MB-231 human BC tumour in SCID mice containing 3.5 × 10^15^ AuNPs (30 nm) complexed to the β-particle emitters, ^177^Lu or ^90^Y or the AE-emitter, ^111^In (Lai et al. 2017). SPECT/CT images from 1 to 168 h of a single NPD incorporating PEG_2K_-*p*Glu(DOTA-[^177^Lu]Lu)_8_-LA_4_-AuNPs inserted into a tumour in a SCID mouse were used to measure the temporal distribution of activity, plot dose maps and derive cumulative dose-volume histograms (DVH) for ^177^Lu, ^90^Y or ^111^In. These doses were simulated by Monte Carlo N-Particle software (MCNP 5, Los Alamos National Security, New Mexico) and voxel-based geometry methods. ^90^Y provided the greatest tumour dose penetration and most homogenous dose distribution (max: 3600 Gy, min: 7.0 Gy) compared to ^177^Lu (max: 11,000 Gy, min: 0.06 Gy) and ^111^In (max: 14,000 Gy, min: 9.8 × 10^–4^ Gy). For ^90^Y, none of the tumor volume received an absorbed dose lower than 0.18 Gy, while for ^177^Lu, 88.5% of the tumor volume fraction received a dose of lower than 0.55 Gy, and for ^111^In, 90.3% of the tumor volume received a dose of lower than 0.73 Gy). Modeling of insertion of 4 NPD into a hypothetical volume (9.6 × 9.6 × 9.6 mm^3^) that incorporated 1.8 MBq, 2.5 MBq or 25.1 MBq of ^90^Y-, ^177^Lu- or ^111^In-labeled AuNPs, respectively with each NPD separated by 4.5 mm in a square formation estimated the maximum doses as 2100 Gy, 6800 Gy and 9300 Gy, respectively. The minimum doses at the volume periphery were 21.8 Gy, 0.12 Gy, and 2.2 × 10^–3^ Gy. Based on this dosimetry, it was concluded that ^90^Y was optimal for assuring the highest and most homogeneous dose distribution in a tumour for a NPD incorporating AuNPs.

### Landscape: radiation dosimetry of radiation nanomedicines

Modeling of the radiation absorbed doses in cancer cells in vitro or in tumours in vivo deposited by radiation nanomedicines provides insight into their potential effectiveness and informs on optimizing their design. Seniwal et al. (2021) calculated cellular S-values using EGSnrc MC code and compared these to MIRDcell for spherical cells and spherical tumours with homogeneously distributed ^198^Au, ^103^Pd or ^153^Sm. Based on the reported data for mangiferin (MCF)-functionalized ^198^AuNP (Katti et al. 2018), AuNP with a ^103^Pd core (Laprise-Pelletier et al. 2017) and cetuximab-conjugated carbon nanocapsules (NCs) incorporating ^153^Sm (Wang et al. 2020b), they modeled the tumour uptake and washout of the NPs injected i.t., then applied a radiobiological model to predict the surviving fraction of tumour cells and the tumour volume post-treatment. Their modeling agreed with experimentally reported results for [^103^Pd]Pd-AuNP and [^153^Sm]Sm-cetuximab carbon NCs but not for [^198^Au]Au-MCF-AuNPs. Finally, they predicted the therapeutic effectiveness under three different scenarios: (i) 0.3 cm^3^ tumour treated i.t. with 20, 40 or 60 MBq of each of the NPs, (ii) 0.3, 0.6 or 1.0 cm^3^ tumour treated i.t. with the same absorbed dose of each of the NPs, and (iii) 0.3 cm^3^ tumour exhibiting various radiosensitivities treated i.t. with the NPs. It was concluded that ^198^Au deposited the highest dose per activity, followed by ^153^Sm and ^103^Pd and for large tumors, ^198^Au and ^153^Sm were more effective than ^103^Pd. This study illustrated that radiobiological models are useful to guide the selection of radionuclides for constructing radiation nanomedicines and selecting the administered activity based on tumour volume, growth rate and radiosensitivity as well as NP tumour uptake and washout rate. Hyrcushko et al. (2011) modeled the absorbed dose in s.c. SCC-4 human HNSCC tumours in nude rats after i.t. infusion of [^186^Re]Re-BMEDA-PEG-liposomes. SPECT/CT images of the tumour were acquired at 0, 2, 4, 20, 44 and 140 h p.i. and were segmented with imaging software (https://mangoviewer.com/) then imported into Matlab software (Mathworks) with voxel sizes of 0.35 × 0.35 × 0.35 mm^3^ for calculation of the time-integrated activity in each voxel. The absorbed dose in each tumor voxel was calculated using the dose-point kernel (DPK) convolution technique, which convolved a matrix of ^186^Re S-values generated with EGSnrc MC simulation code with a matrix of time-integrated activities from SPECT/CT. The effective uniform dose (EUD) defined as the uniform value of biologically effective dose (BED) was calculated for each tumour based on the linear-quadratic model, voxel BED, repopulation and repair time constants. Dose-volume histograms (DVHs) were calculated from the voxel absorbed doses and demonstrated large heterogeneity of intratumoral doses (0–2000 Gy). The correlation between the average tumour absorbed dose (392–982 Gy) and tumour response was poor (R^2^ = 0.22) but the correlation between EUD (6.10–26.99 Gy) and tumour response was excellent (R^2^ = 0.84). This study showed that an EUD model incorporating dose heterogeneity in a tumour would be helpful in predicting responses from radiation nanomedicines. Medina et al. (2007) described a method to visually and digitally show the spatial dose distribution in a tumour following i.t. injection of [^186^Re]Re-BMEDA-liposomes (122 nm) in rats with s.c. SCC-4 human head and neck squamous cell carcinoma tumours. Static planar γ-camera images were obtained immediately after injection and at 3 h p.i. These showed high uptake in the tumour (45–50% ID/g) but heterogeneous intratumoural distribution. The rats were sacrificed, the tumours excised and sectioned into 3 mm thick slices and these were then exposed to HS Gafchromic film at 4 °C for 18 h. The optical density of the Gafchromic film was correlated with the absorbed dose and calibrated by exposing the films to known dose rates from a ^60^Co source. Dose maps for each tumour section were obtained by scanning the exposed films and the DVH calculated for each tumour.

Emfietzoglou et al. (2001) compared the dosimetry of the β-particle emitters, ^67^Cu, ^188^Re, ^90^Y or ^131^I incorporated into liposomes and injected i.v. into mice with intramuscular Ehrlich ascites tumours or intrahepatic C26 colon cancer tumours. These radionuclides were selected to include a range of physical half-lives (0.71–8.0 d) and mean β-particle energies (0.141–0.935 MeV). Different forms of liposomes were considered based on the published biodistribution data: (i) small unilamellar vesicles (SUV; < 100 nm); (ii) multi-lamellar vesicles (MLV; > 1000 nm); (iii) SUV coated with monosialoganglioside (G_MI_); and iv) SUV coated with PEG. The biodistribution in mice (%ID/organ) was scaled to a human using the ratio of human organ weight/human body weight vs. mouse organ weight/mouse body weight. It was assumed that the tumour in a human was ~ 280 g (spherical 8 cm diameter lesion). Red marrow activity was derived from blood data assuming that the ratio of liposomes concentration in red marrow versus the blood was 0.36 (Sgouros 1993). The MIRD schema was used to calculate the average doses in the human for the tumour, liver, spleen, kidneys, lungs, red marrow and total body. The dosimetry projected that SUV incorporating ^90^Y were optimal, since administration of 13.4 GBq to a human would deliver an effective tumour dose (20 Gy) and tolerable doses in the liver (25 Gy) and red marrow (1.4 Gy). However, these doses in the liver and red marrow approach the maximum tolerated doses of radiation for these tissues (30–32 Gy for liver and 2–5 Gy for red marrow) (Wahl et al. 2021), again indicating the challenge of delivering effective tumour doses while avoiding toxicity on normal tissues for i.v. injected radiation nanomedicines. This same group (Emfietzoglou et al. 2005) estimated the dosimetry of the β-particle emitters, ^32^P, ^90^Y, ^188^Re, ^67^Cu or ^131^I or AE-emitters, ^123^I or ^125^I incorporated into SUV (50–100 nm) or MLV (80–1000 nm) liposomes modified with anti-PSMA J591 antibodies and incubated in vitro with LNCaP human prostate cancer spheroids (200 μm diameter). Regardless of the liposomal form, high energy β-emitters (e.g. ^90^Y) provided more homogeneous absorbed doses in the spheroids vs. low energy AE-emitters (e.g. ^123^I). These results agree with our reported dosimetry modeling of tumours implanted i.t. with a NPD incorporating AuNPs complexed to the β-particle emitters, ^177^Lu or ^90^Y or the AE-emitter, ^111^In, where the higher energy β-particles emitted by ^90^Y provided the most homogeneous dose distribution (Lai et al. 2017).

#### Nanoscopic and microscopic dose deposition

As discussed earlier, interaction of X-rays with AuNPs causes release of secondary AE and PE that enhance the RBE of XRT for killing cancer cells, i.e. dose-enhancement effect. Emission of α-particles, β-particles or AE by radionuclides may also interact with dense inorganic NPs to produce secondary AE and PE that enhance dose deposition and increase the RBE on the nanoscopic or microscopic scale. Gholami et al. (2019) reported a dose simulation study which examined the interaction of α-particles emitted by ^213^Bi or ^223^Ra or β-particles emitted by ^67^Cu, ^177^Lu or ^90^Y with spherical Fe_2_O_3_ nanoparticles (IONPs). These radionuclides were distributed in a 1 nm wide shell surrounding the 6 nm core of the IONPs. A cluster of 500 IONPs was distributed in the center of a water phantom cube (3.5 × 3.5 × 3.5 μm^3^) and the separation distance between IONPs varied from 1 to 50 nm. The study showed that at a separation distance of 1 nm, there was 20.7%, 19.3%, 18.3%, 17.6% and 16.7% increased dose for IONPs bound to ^223^Ra, ^213^Bi, ^90^Y and ^67^Cu vs. radionuclides without IONPs, respectively. The dose-enhancement effect decreased as separation distance increased. Dose-enhancement was due to the release of secondary electrons from the IONPs following interaction with the α- or β-particles emitted by the radionuclides. Maschmeyer et al. (2020) conduced a MC dose-modeling study to estimate the nano-Radio Enhancement Ratio (nano-RER) defined as the ratio of the number of secondary electrons produced with/without 5 nm superparamagnetic iron oxide NPs (SPIONs) bound to ^67^Cu. The nano-RER for a single [^67^Cu]Cu-SPION decreased dramatically as the distance from the SPION increased. The highest nano-RER was 1.15 (i.e. 15% increase in secondary electrons) which occurred within 100 nm of the SPION surface. Clustering of the SPIONs and larger SPION clusters increased the nano-RER, but the nano-RER was decreased as the separation distance between the clusters increased.

The effect of the AE emissions from ^125^I, ^111^In or ^99m^Tc encapsulated in nanoshells or coated on spherical NPs composed of Fe, Ag, Gd, Au or Pt was modeled by Sung et al. (2016). Doses were estimated for a water phantom using MC simulation with Geant4-Penelope code. The inner/outer diameters of the nanoshells or the diameter of nanospheres were varied to determine the effect of NP size on dose-enhancement vs. radial distance from the NP surface. The nanoshells encapsulating these radionuclides stopped low energy electrons and decreased the number of electrons emitted at the NP-water interface. Thus, the total dose per decay at the same radial distance was highest for a nanosphere, followed by no NP, then by a nanoshell. For nanoshells encapsulating these radionuclides, the dose-enhancement was < 1 at a radial distance < 10 nm but peaked at ~ 20 nm for most studied combinations. In contrast, for nanopsheres coated with these radionuclides the dose-enhancement was highest at the NP-water interface, then decreased dramatically within 5 nm from the surface of the NP. The local microscopic doses at radial > 100 nm for NP were not larger than without NP. This study concluded that AE therapy combined with NP could potentially provide better therapeutic effect than AE alone, however, the choice of AE emitters and the design of NP should be optimized based on the distance between AE emitters and biological targets. Hahn et al. (2021) estimated the absorbed dose in various subcellular compartments (cell membrane, cytoplasm, nucleus and mitochondria) of Chinese hamster ovary (CHO) cells by ^198^AuNPs located either randomly in cytosol or in cell nucleus by MC methods using Geant4 code combined with the Topas and TsSphericalCellSphericalNP model and two AuNP distribution models, simplified continuous Au or discrete AuNP (5 nm)-geometric distribution. The purpose of the study was to compare the effect of AuNP distribution models on the calculated dose enhancement by AuNP and to understand the factors such as locations of AuNPs and the % mass of Au in the cell influencing the dose enhancement of AuNP. The continuous model underestimated the energy deposition per decay of ^198^Au in most compartments in the cells by 4–15% except cell membrane. For both models, the energy deposited per decay in cell organelles increased as the mass Au % increased. Increasing the number of AuNPs targeted to the nucleus increased the energy deposited in the whole cell and the radiosensitive nucleus, but decreased the energy deposited in the cytoplasm, mitochondria and cell membrane compared to AuNPs randomly distributed in the cytoplasm. This study helped to guide the design of targeted ^198^AuNP for more effective cancer therapy.

### Journey: ^177^Lu-Labeled NPs for treatment of GBM

**Fig. 9:**
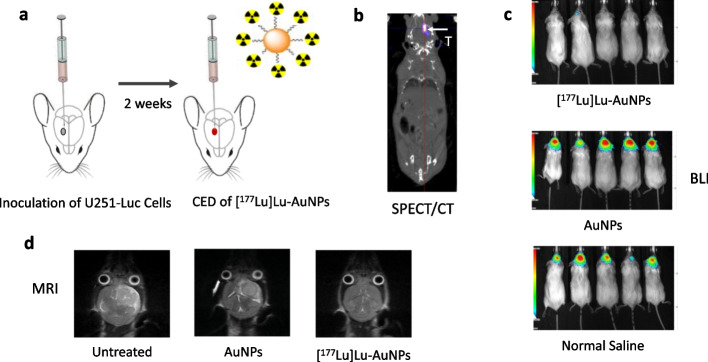
**a** Experimental design for studies of [^177^Lu]Lu-AuNPs for treatment of human GBM tumours. Orthotopic human GBM tumours were established in the brain of NRG mice by stereotaxic inoculation of luciferase-transfected U251-Luc cells. At two weeks post-inoculation, [^177^Lu]Lu-AuNPs (1–2 MBq; 4 × 10^11^ AuNPs AuNPs) were infused by CED into these tumours. **b** SPECT/CT images at 14 d post-infusion of [^177^Lu]Lu-AuNPs showed retention of activity at the site of infusion in the brain with no redistribution within the brain or to organs outside the brain. **c** Bioluminescence imaging (BLI) at 21 d post-treatment with the radiation nanomedicine showed no evidence of tumours in the brain in 4/5 mice with one mouse exhibiting a small BLI signal in the brain, while all mice treated with unlabeled AuNPs or receiving normal saline exhibited a strong BLI signal in the brain. **d** MRI at 28 d post-treatment in representative mice showed no evidence of tumour in the brain for mice treated with the radiation nanomedicine, but large GBM tumours in the brain in control mice receiving unlabeled AuNPs or normal salie. Adapted and reprinted by licence from: Georgiou, C.J. et al. 2023. Mol. Pharm. 20, 582–592

A promising application of locally-administered radiation nanomedicines is treatment of glioblastoma multiforme (GBM) since metastasis of GBM outside the brain is very rare. GBM is the most common and most lethal form of brain cancer. GBM is currently treated by surgery, external radiation and temozolomide chemotherapy (Stupp et al. 2005). However, almost all patients develop recurrence, often within 2 cm of the surgical margins. Recurrent GBM is very difficult to treat and consequently, the median survival of patients with GBM is < 18 months (Delgado-Lopez and Corrales-Garcia 2016). Local infusion of radiation nanomedicines by convection enhanced delivery (CED) into the surgical cavity post-resection may eradicate residual tumour at the margins and prevent recurrence, potentially improving survival. We studied a radiation nanomedicine for treatment of GBM by stereotaxically infusing 1.1 MBq [^177^Lu]Lu-MCP-AuNPs (~ 23 nm; 4 × 10^11^ AuNPs) by CED into small (~ 2 mm) luciferase-transfected U251-Luc human GBM tumours in the brain in NRG mice (Georgiou et al. 2023) (Fig. 9a). SPECT/CT imaging up to 21 d p.i. showed retention of activity at the i.t. infusion site with minimal redistribution to other regions of the brain or to organs outside the brain (Fig. 9b). Biodistribution studies revealed that activity in the tumour-involved hemisphere was 200–300% ID/g, while activity in the normal brain was only 1–2% ID/g and in other organs including the liver and spleen was < 3% ID/g. The retention of [^177^Lu]Lu-MCP-AuNPs in the brain is believed to be due to an “anchoring” effect of the AuNPs, due to the bidirectional nature of the blood–brain barrier (D'Amico et al. 2021) the large AuNP construct was unable to cross into systemic circulation, while [^177^Lu]Lu-MCP not bound to AuNPs was rapidly eliminated from the brain within 72 h post-infusion. The radiation absorbed dose in the tumour was 600 Gy while the doses to the surrounding brain were 100-fold lower (6.4 Gy) and to the contralateral hemisphere of the brain were 2000-fold lower (0.3 Gy). Doses to organs outside the brain were < 0.2 Gy, including in the liver and spleen. Treatment of NRG mice U251-Luc tumours with [^177^Lu]Lu-MCP-AuNPs achieved almost complete tumour growth arrest demonstrated by bioluminescence imaging (BLI) (Fig. 9c) or MRI (Fig. 9d) while tumours in control mice treated with unlabeled AuNPs or normal saline grew rapidly. No visible tumour was found at 28 d p.i. of [^177^Lu]Lu-MCP-AuNPs with the treatment evaluated by MRI and by ex vivo histological staining. All normal saline treated mice died within 39 d and mice treated with unlabeled AuNPs died within 45 d, but 5/8 mice treated with [^177^Lu]Lu-MCP-AuNPs survived up to 150 d. There was no apparent normal tissue toxicity including to the brain. These results are very promising for CED of radiation nanomedicines for treatment of GBM.

### Landscape: radiation nanomedicines for GBM

No other studies of radiolabeled AuNPs for treatment of GBM have been reported, but liposomal NPs labeled with the β-particle emitters, ^186^Re or ^188^Re have been investigated. Cikankowitz et al. (2017) studied [^188^Re]Re-nanocapsules (NCs) administered by CED for treatment of orthotopic human Lab1 GBM tumours in nude NMRI mice. Activity was retained (> 70%) in the tumour and peritumoural space with no redistribution to the contralateral brain hemisphere. Treatment of mice with Lab1 tumours with two amounts (3 MBq each) of [^188^Re]Re-NCs separated by 7 d increased median survival to 56 d versus 20–21 d for mice receiving unlabeled NCs or no treatment. Moreover, radiation nanomedicine treatment decreased tumour volume by > 50% at 18 d post-CED compared to mice treated with unlabeled NCs. There was no toxicity observed on the normal brain. This same group (Vanpouille-Box et al. 2011) studied [^188^Re]Re-NCs (two amounts of 2.8 MBq separated by 6 d) administered by CED for treatment of 9L rat glioma tumours in rats achieving cure rates of > 80%, which may have been due to a combined radiation and immune response, since immune cells were found infiltrating the tumour. Median survival was increased to > 120 d in rats treated with [^188^Re]Re-NCs versus < 30 d in untreated rats. The greater effectiveness of CED for radiation nanomedicines to treat GBM is illustrated through a study by Huang et al. (2015) who treated Fisher rats with orthotopic F98 glioma tumours with i.v. injected [^188^Re]Re-liposomes at much higher amounts (333 MBq). Tumour uptake was only 2% ID/g and median survival was only slightly increased to 20 d versus 18 d for untreated rats. Body weight decreased indicating normal tissue toxicity. Séhédic et al. constructed [^188^Re]Re-loaded lipid nanocapsules (LNCs) modified with 12G5 antibodies to target C-X-C chemokine receptor type 4 (CXCR4), a receptor involved in GBM tumour growth and invasiveness mediated by matrix metalloproteinases (MMPs) (Zhang et al. 2005). Treatment of SCID mice with orthotopic U87MG CXCR4-positive human GBM tumours with 2.7 MBq of [^188^Re]Re-12G5-LNCs increased median survival to 74 d versus 38 d for untreated mice or 34 d for mice treated with unlabeled and non-targeted LNCs. Non-targeted [^188^Re]Re-LNCs were also effective for treating these GBM tumours. Philips et al. (Phillips et al. 2012) examined the effect of infusion volume of [^186^Re]Re-liposomes administered by CED in rats with orthotopic luciferase-transfected U-87 MG-luc2 human GBM tumours. Large infusion volumes (100 μL) were associated with redistribution to the brainstem and cerebellum, while small volumes (25 μL) were retained in the tumour for up to 96 h. Absorbed doses as high as 1800 Gy were deposited in GBM tumours by CED of up to 4.6 MBq of [^186^Re]Re-liposomes. There was a major treatment response with almost complete elimination of tumour detected by BLI or MRI. Median survival was increased to 126 d for rats treated with the radiation nanomedicine versus 49 d for untreated rats. There was no apparent normal tissue toxicity. Single administration of [^186^Re]Re-liposomes by CED was evaluated in a Phase I/IIA dose escalation trial in patients with recurrent GBM which found that injected activities up to 1535 MBq (41.5 mCi) were safe and without dose-limiting toxicities (Brenner et al. 2023).

Schultz et al. constructed metallofullerenes labeled with ^177^Lu (Shultz et al. 2011). CED of ^177^Lu-labeled metallofullerenes (0.25–1.35 MBq) into orthotopic U87MG tumors in nude mice improved median survival with > 80% of mice treated with 1.35 MBq surviving > 100 d, while untreated mice survived < 29 d. Moreover, tumours in treated mice were smaller assessed by ex vivo histological staining (Wilson et al. 2012). Together, the results of these studies confirm our findings (Georgiou et al. 2023) that radiation nanomedicines administered by CED deposit high absorbed doses in GBM tumours in animal models that result in strong tumour growth-inhibitory effects, but are not toxic to normal tissues including normal brain, due to the restricted diffusion within the brain or redistribution to organs outside the brain mediated by the anchoring effects of NPs.

### Landscape: NPs labeled with α-particle emitting radionuclides

Radionuclides that emit α-particles are very attractive for incorporation into NPs because several MeV of energy is released during α-decay. This energy is deposited over a short range (50–100 μm) resulting in high LET (50–230 keV/μm) that is extremely potent for killing cancer cells (Aghevlian et al. 2017). In contrast, β-particles deposit their energy over several mm range in tissues, resulting in low LET (< 0.1 keV/μm) which is less effective for damaging DNA in cancer cells. Moreover, α-particles may directly inflict lethal DNA DSBs while β-particles rely on indirect DNA damage mediated by reactive oxygen species (ROS). Thus, α-particles may be more effective than β-particles for treating hypoxic tumours (Aghevlian et al. 2017).

#### Actinium-225

^225^Ac (t_1/2_ = 10 d) is an α-particle emitting radionuclide that decays through a series of daughter products that are themselves α-particle or β-particle emitters to stable ^209^Pb. Most studies of radiation nanomedicines incorporating α-particle emitters have used liposomes. Chang et al. (2008) actively loaded ^225^Ac into PEGylated liposomes (~ 121 nm) by complexing ^225^Ac with the ionophores, oxine or A23187 at 65 °C and then mixing these complexes with liposomes. Loading efficiency for ^225^Ac was high (up to 60–80%) but there was rapid and extensive release of ^225^Ac (50–80%) in vitro within 2 h at 37 °C in PBS or medium containing serum. This instability would make these liposomal formulations not feasible for therapy studies. Bandekar et al. (2014) incorporated ^225^Ac into PEGylated liposomes (~ 107 nm) modified with anti-PSMA J591 antibodies or A10 aptamer. These liposomes incorporated DOTA to strongly complex ^225^Ac. Loading was achieved at 65–67 °C by incubating liposomes with ^225^Ac complexed to A23187 ionophore with transchelation of ^225^Ac within the liposome to DOTA. Loading efficiency ranged from 58 to 85%. These liposomes were relatively stable, retaining ~ 70% of ^225^Ac in vitro up to 24 h when incubated at 37 °C in medium containing serum. J581-modified liposomes exhibited greater binding to PSMA-positive LnCAP human and Mat-Lu rat prostate cancer than A10-modified liposomes. There was no binding to PSMA-negative cell lines. [^225^Ac]Ac-DOTA-J591-liposomes were more cytotoxic than [^225^Ac]Ac-A10-liposomes in vitro against LNCap and Mat-Lu cells, decreasing cell viability to 10–20% after incubation for 24 h at up to 0.4 MBq/mL. Non-targeted [^225^Ac]Ac-DOTA-liposomes were also cytotoxic on PSMA-positive cells and targeted [^225^Ac]Ac-DOTA-J591-liposomes and [^225^Ac]Ac-DOTA-A10-liposomes demonstrated cytotoxicity towards PSMA-negative cells, but these effects were lower than for targeted liposomes on PSMA-positive cells. Sofou et al. (2007) synthesized PEGylated multivesicular liposomes (MUVELs) composed of large liposomes (~ 400 nm) that contained entrapped small vesicles (~ 100 nm) to aid in retention of ^225^Ac daughters and limit normal tissue toxicity. ^225^Ac complexed to DOTA was passively loaded into the liposomes during synthesis. The efficiency of loading was low (< 10%) but ^225^Ac was stably retained (> 98%) over 30 d by these MUVELs. Unfortunately, there was release of the ^213^Bi daughter of ^225^Ac (70%). These [^225^Ac]Ac-DOTA-MUVELs were further modified with trastuzumab and showed specific binding and internalization by HER2-positive SKOV3-NMP2 human ovarian cancer cells. This same group compared cationic and zwitterionic PEGylated liposomes of different sizes (~ 180, 400 or 600–650 nm) for retaining ^225^Ac and its daughters (Sofou et al. 2004). It was theorized that if the liposome dimension was sufficiently large, it would retain ^213^Bi and other ^225^Ac daughters, particularly as these are ionic in nature and would not be able to escape by penetrating the liposomal lipid membrane. ^213^Bi retention was greater for 600 nm versus 100 nm zwitterionic liposomes. Nonetheless, ^213^Bi retention was very low (< 12%) for zwitterionic or cationic liposomes. ^225^Ac was retained better in zwitterionic liposomes (> 90%) than in cationic liposomes (75–85%).

A challenge in using NPs incorporating α-particle emitters for cancer therapy is the short range of α-particles (50–100 μm) since not all tumour cells within a lesion may be irradiated due to the limited penetration of NPs into tumours (Lee et al. 2010). Zhu et al. (2017) aimed to address this challenge by constructing pH-responsive liposomes that incorporated ^225^Ac complexed to DOTA that would release their payload at the slightly acidic pH of the tumour microenvironment. They treated small (200 μm) or large (400 μm) spheroids composed of HER2-positive human BC cells in vitro with trastuzumab-modified pH-responsive [^225^Ac]Ac-DOTA-liposomes or non-pH responsive [^225^Ac]Ac-DOTA-liposomes. While the liposomal carrier remained mostly at the surface of the spheroids, only pH-responsive liposomes showed uniform ^225^Ac activity diffusion within the spheroids, while non-pH responsive liposomes showed low activity in the centre and higher activity on the surface. DNA DSBs measured by γ-H2AX immunofluorescence were predominant near the surface for all liposomes, but there was higher γ-H2AX intensity near the centre of the spheroid for pH-responsive [^225^Ac]Ac-DOTA-liposomes. In addition, pH-responsive [^225^Ac]Ac-DOTA-liposomes were more effective than non-pH responsive liposomes for inhibiting the growth of HER2-positive BT-474 and HER2-negative MDA-MB-231 spheroids. In mice with s.c. HER2-negative MDA-MD-231 tumours, i.v. injection of 9.25 kBq of pH-responsive [^225^Ac]Ac-DOTA-liposomes inhibited tumour growth and prolonged survival, whereas non-pH responsive liposomes were not effective. However, the MTD of these non-targeted [^225^Ac]Ac-DOTA-liposomes after i.v. injection in healthy mice was 14.8 kBq, causing pneumonitis and decreased spleen size, although there was no renal or liver toxicity.

Inorganic NPs have been loaded with ^225^Ac to construct radiation nanomedicines. McLaughlin et al. (2014) synthesized NPs composed of ^225^Ac embedded in lanthanum (La) and gadolinium phosphate (GdPO_4_) layers that were then coated with a gold (Au) shell. These NPs were further modified with antibodies that bind murine thrombomodulin expressed in lung endothelium. [^225^Ac]Ac–La–GdPO_4_–Au NPs localized in the lungs of mice and retained the ^213^Bi daughter in lung tissue, suggesting that these NPs were stable and did not release the daughter products of ^225^Ac. Treatment of mice with murine EMT-6 mammary carcinoma metastases in the lungs by i.v. injection of 44 kBq of [^225^Ac]Ac–La–GdPO_4_–Au NPs reduced the number of EMT-6 lung colonies (metastases) by fourfold compared to untreated mice. Karpov et al. (2022) incorporated ^225^Ac into silica (SiO_2_) NPs (~ 100 nm) that were then surface-coated with titanium doxide (TiO_2_) or gold (Au) to aid in the retention of ^225^Ac and its daughters. Uncoated [^225^Ac]Ac–SiO_2_ NPs released 70% of ^225^Ac by 30 d when incubated in human serum, whereas [^225^Ac]Ac–SiO_2_–TiO_2_ NPs and [^225^Ac]Ac–SiO_2_–Au NPs released only 0.3% and 2.6%, respectively. The release of ^221^Fr and ^213^Bi daughters of ^225^Ac at 30 d was 58% and 46%, respectively for uncoated [^225^Ac]Ac–SiO_2_ NPs, but this was reduced to 15% and 5%, respectively for coated [^225^Ac]Ac–SiO_2_–TiO_2_ NPs and to 20% and 12%, respectively for [^225^Ac]Ac–SiO_2_–Au NPs. Administration of 74 kBq i.v. of uncoated [^225^Ac]Ac–SiO_2_ NPs caused lung toxicity in mice, but this was not discovered for [^225^Ac]Ac–SiO_2_–TiO_2_ or [^225^Ac]Ac–SiO_2_–Au NPs. Further, slight histological changes were found in the liver for [^225^Ac]Ac–SiO_2_ NPs but not for [^225^Ac]Ac–SiO_2_–TiO_2_ or [^225^Ac]Ac–SiO_2_–Au NPs. Renal toxicity is a reported concern for the released ^213^Bi daughter of ^225^Ac, and severe inflammation was noted in the kidneys of mice injected with uncoated [^225^Ac]Ac–SiO_2_ NPs but this was not observed for [^225^Ac]Ac–SiO_2_–TiO_2_ or [^225^Ac]Ac–SiO_2_–Au NPs. These results suggest that coating [^225^Ac]Ac–SiO_2_ NPs with TiO_2_ or Au shells retains ^225^Ac and its daughters within the NPs, reducing normal tissue toxicity.

Single walled carbon nanotubes (SWCNTs) are nanometer-sized cylindrical structures composed of fullerene graphine (Ajayan 1999). Matson et al. (2015) loaded ^225^Ac^3+^ with/without co-loading of gadolinium (Gd^3+^) ions into 20–80 nm long SWCNTs. Loading efficiency for ^225^Ac^3+^ was very high (> 95%) while co-loading of ^225^Ac^3+^ and Gd^3+^ was only 50% due to competition with Gd^3+^ ions. However, co-loading of ^225^Ac^3+^ and Gd^3+^ provided greater stability to release of ^225^Ac when incubated for 2 h at 37 °C in serum (80% vs. 40%), which was hypothesized to be due to formation of Ac-Gd clusters within the side-wall defects in the SWCNTs. No cytotoxicity studies in vitro or tumour therapy studies in vivo were conducted. Mulvey et al. (2013) constructed 350 nm length SWCNTs (diameter ~ 1.2 nm) conjugated to DOTA complexed to ^225^Ac or ^111^In that were also modified with complementary morpholino oligonucleotides (cMORF) for pre-targeted delivery to tumours that bound MORF-conjugated anti-CD20 rituximab. To assess the toxicity of the [^225^Ac]Ac-DOTA-cMORF-SWCNTs, increasing amounts (16.6–100 kBq) were administered i.v. to healthy Balb/c mice. Dose-dependent bone marrow toxicity was found for mice injected with 50–100 kBq and amounts > 83 kBq caused death. Thus, treatment studies were performed in SCID mice with CD20-positive intraperitoneal (i.p.) Daudi B-cell lymphoma tumours. The mice were administered rituximab-MORF, followed 24 h later with 12.3–36.9 kBq of [^225^Ac]Ac-DOTA-cMORF-SWCNTs. BLI demonstrated complete eradication of Daudi tumours in mice receiving 24.6 or 36.9 kBq of [^225^Ac]Ac-DOTA-cMORF-SWCNTs, while control mice injected with normal saline or unlabeled DOTA-cMORF-SWCNTs exhibited rapid tumour growth. Mice pre-targeted with an irrelevant anti-CD33-MORF followed by [^225^Ac]Ac-DOTA-cMORF-SWCNTs exhibited a transient tumour response but subsequently rapidly progressed. Ruggiero et al. (2010) synthesized 200–1000 nm length SWCNTs (diameter 1.4 nm) modified with DOTA to complex ^225^Ac or with desferrioxamine (DFO) for labeling with ^89^Zr. These SWCNTs were further modified with E4G10 antibodies to target the tumour neovasculature. Athymic mice with s.c. LS174T human colon cancer xenografts were treated with 16.1 kBq of [^225^Ac]Ac-DOTA-E4G10-SWCNTs by i.v. injection. PET was performed in mice with s.c. LS174T tumours injected i.v. with 4.2 MBq of [^89^Zr]Zr-DFO-E4G10-SWCNTs. PET showed high liver and kidney uptake and low tumour uptake (~ 0.5% ID/g at 24 h p.i.). Nonetheless, high specific activity (851 GBq/g) of [^225^Ac]Ac-DOTA-E4G10-SWCNTs transiently inhibited tumour growth and prolonged survival compared to mice treated with irrelevant anti-KLH-modified SWCNTs or no treatment. Normal tissue toxicity was not evaluated. Despite some promising results for ^225^Ac-labeled SWCNTs as radiation nanomedicines, it should be recognized that unmodified CNTs have been found to be cytotoxic against many normal cell types, due to their water-insolubility which limits their elimination (Reilly 2007). Functionalization of SWCNTs to increase their water-solubility greatly reduces their cytotoxicity and promotes blood clearance.

#### Other α-particle emitters

^213^Bi (t_1/2_ = 46 min) is a daughter radionuclide of ^225^Ac that decays by β-particle emission (98%; E_βmax_ = 0.444 MeV) to ^213^Po (t_1/2_ = 4.2 µs) which then decays by α-particle emission (E_α_ = 8.4 MeV) to ^209^Pb (t_1/2_ = 3.25 h). ^209^Pb decays by β-particle emission (E_βmax_ = 0.198 MeV) to stable ^209^Bi. Alternatively, ^213^Bi decays by α-particle emission (2%; E_α_ = 5.5–5.9 MeV) to ^209^Tl (t_1/2_ = 2.2 min) which decays to ^209^Pb by β-particle emission (E_βmax_ = 0.659 MeV) to stable ^209^Bi. Lingappa et al. (2010) incorporated ^213^Bi complexed to CHX-A′′-DTPA into PEGylated liposomes (100 nm) that were modified with HER2/neu antibodies. [^213^Bi]Bi-CHX-A′′-DTPA-anti-HER2-liposomes were more cytotoxic than [^213^Bi]Bi-CHX-A′′-DTPA-liposomes in vitro against HER2/neu–expressing NT2.5 murine mammary carcinoma cells. Biodistribution studies in neu-N transgenic mice with metastatic NT2.5 tumours at 4 h post i.v. injection of [^213^Bi]Bi-CHX-A′′-DTPA-anti-HER2-liposomes revealed high uptake in the spleen (40% ID/g) but relatively low uptake (< 5% ID/g) in other tissues. Tumour uptake was maximal at 0.5 h p.i. (3% ID/g) but decreased at later time points. Hematopoetic toxicity was the major dose-limiting toxicity for [^213^Bi]Bi-CHX-A′′-DTPA-anti-HER2-liposomes. Treatment of mice with metastatic NT2.5 tumours with 19.2 MBq of [^213^Bi]Bi-CHX-A′′-DTPA-anti-HER2-liposomes improved the median survival to 38 d versus 29 d for untreated mice and 27 d for mice treated with unlabeled liposomes. ^212^Pb (t_1/2_ = 10.6 h) decays by β-particle emission (E_βmax_ = 0.57 MeV) to ^212^Bi (t_1/2_ = 60.6 min) which then decays to ^212^Po (t1/2 = 0.3 μs) by β-particle emission (64%; E_βmax_ = 2.2 MeV). ^212^Po then decays by α-particle emission (E_α_ = 8.8 MeV) to stable ^208^Pb. Alternatively, ^212^Bi decays by α-particle emission (36%; E_α_ = 6.1 MeV) to ^208^Tl (t_1/2_ = 3.0 min) which decays to stable ^208^Pb by β-particle emission (E_βmax_ = 1.8 MeV). Thus, ^212^Pb is an attractive radionuclide for incorporation into NPs due to its multiple α- and β-particle emissions. Pikul et al. (1987) incorporated ^212^Pb complexed to dextran into large unilamellar liposomes (350–500 nm). Treatment of B16F10 murine melanoma cells in vitro with [^212^Pb]Pb-dextran-liposomes decreased their survival by 85% with as few as 5–7 α-particles traversing the cells. ^211^At (t_1/2_ = 7 h) decays through ^211^Po and ^207^Bi daughter products to stable ^207^Pb, emitting α-particles (Eα = 5.9–7.4 MeV). Kato et al. (2021) constructed PEGylated [^211^At]At-labeled AuNPs and injected these i.t. in rats with s.c. C6 glioma or mice with s.c. PANC-1 human pancreatic cancer xenografts. Activity was strongly retained in these tumours with no apparent redistribution to other tissues. Smaller AuNPs (30 nm) diffused more readily and homogeneously within tumours than larger (120 nm) AuNPs. Treatment of tumour-bearing rats with 1.4 MBq or mice with 1.2 MBq of [^211^At]At-PEG-AuNPs arrested tumour growth without causing toxicity, assessed by monitoring body weight. Mean tumour size at 40 d post-treatment was tenfold lower for rats or mice receiving [^211^At]At-PEG-AuNPs than in untreated mice.

^223^Ra (t_1/2_ = 11.4 d) decays through several daughter radionuclides (^219^Rn, ^215^Po, ^211^Pb, ^211^Bi, ^211^Po and ^207^Tl) to stable ^207^Pb, by emitting 4 α-particles (E_α_ = 5.0–7.5 MeV) as well as by emission of several β-particles. ^223^RaCl_2_ (Xofigo; Bayer) is approved for treatment of painful bone metastases in patients with castration-resistant prostate cancer and is the only α-particle emitting therapeutic agent in routine clinical use (McGann and Horton 2015). Since there are limited chelators available to complex radium to biomolecules (Henriksen et al. 2002), incorporating ^223^Ra into NPs that are surface-modified with tumour targeting vectors could provide a route to selective delivery of ^223^Ra to tumours. Gaweda et al. (2020) synthesized spherical ^223^Ra barium ferrite NPs ([^223^Ra]BaFeNPs) that were further modified with trastuzumab via a 3-phosphonopropionic acid (CEPA) linker to bind to HER2. Based on dynamic lght scattering (DLS) analysis, trastuzumab conjugation increased the hydrodynamic diameter of BaFeNPs in PBS from 116 to 218 nm. [^223^Ra]BaFeNPs retained > 97–98% of ^223^Ra and 88% and 94% of the ^211^Bi and ^211^Pb daughters, respectively, in human serum up to 30 days. [^223^Ra]BaFeNPs were specifically bound, internalized and localized in the nucleus of HER2-positive SK-OV-3 human ovarian cancer cells. [^223^Ra]BaFeNPs were more cytotoxic to SK-OV-3 cells and SK-OV-3 spheroids in vitro than ^223^RaCl_2_ or non-targeted [^223^Ra]BaFeNPs. Zeolites are crystalline microporous aluminosilicates, that include zeolite A usually synthesized as the Na^+^ form [Na_12_Al_12_Si_12_O_48_ ≤ 27 H_2_O; NaA]. Czerwinska et al. (2020) constructed NaA nanozelite NPs (120 nm) labeled with ^223^Ra by exchanging ^223^Ra with Na^+^. These NPs were further modified with silane-PEG-NH_2_ to enable conjugation to N-hydroxysuccinimide (NHS)-derivatized anti-PSMA monoclonal antibodies D2B. [^223^Ra]RaA-silane-PEG-D2B NPs retained > 95% of ^223^Ra and its daughters, ^211^Bi and ^211^Pb up to 12 days in 0.9% NaCl and human serum. [^223^Ra]RaA-silane-PEG-D2B NPs were cytotoxic in vitro to DU-145 and LNCaP human prostate cancer cells. However, i.v. injection of [^223^Ra]RaA-silane-PEG-D2B NPs in LNCAP tumor-bearing nude mice, resulted in high uptake in the liver, spleen, lungs, and bone (Lankoff et al. 2021). Hematopoietic and liver toxicity were limiting factors. Thus, it was concluded that i.v. administration of [^223^Ra]RaA-silane-PEG-D2B NPs was not feasible. However, local administration of [^223^Ra]RaA-silane-PEG-D2B NPs may overcome these limitations, but was not studied.

### Landscape: future clinical translation of radiation nanomedicines

**Fig. 10:**
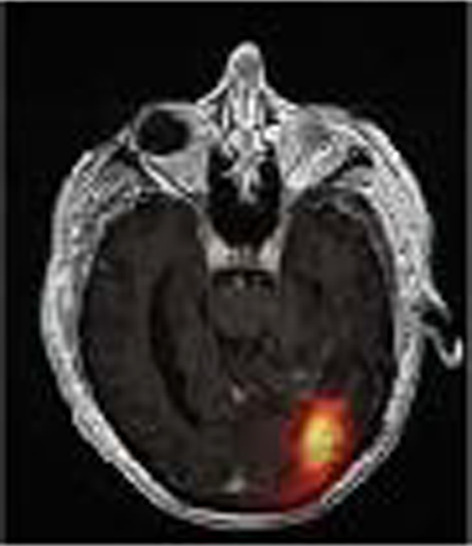
Localization of ^188^Re-labeled nanoliposomes post-infusion by CED in a patient with GBM. The activity remains at the site of infusion and does not redistribute to other areas of the brain. Adapted and reprinted with permission from: Woodall, R.T. et al. Patient specific, imaging-informed modeling of rhenium-186 nanoliposome delivery via convection enhanced delivery in glioblastoma multiforme. Biomed. Phys. Eng. Express. 2021;7(4): 10.1088/2057–1976/ac02a6

Several nanomedicines for delivery of chemotherapeutic drugs have completed clinical trials and were approved for treatment of cancer (Thapa and Kim 2023). However, to date no radiation nanomedicine has been approved for cancer treatment. Nonetheless, ^99m^Tc-sulfur colloid is a radiolabeled NP (particle size ~ 300 nm) that is injected i.v. and has been used routinely for decades in nuclear medicine for imaging the liver, spleen and bone marrow (Geslien et al. 1976). More recently, filtered ^99m^Tc sulfur colloid with a smaller particle size (~ 100 nm) injected interstitially has been used to detect the sentinel lymph node in patients with breast cancer (Giammarile et al. 2013). Some other radiolabeled NPs have been studied clinically for tumour imaging. Lee et al. (2017) investigated PET imaging with a ^64^Cu-labeled liposomal drug delivery system (MM-302) to probe the EPR properties of tumours in patients with metastatic breast cancer, which may predict response to these NPs incorporating chemotherapeutic drugs. Nonetheless, these are examples of radiolabeled NPs for imaging purposes and not for radiotherapeutic applications. The challenge to advancing radiation nanomedicines for cancer treatment is sequestration by the liver and spleen and low tumour uptake after i.v. injection which results in a low therapeutic index as revealed in many preclinical studies discussed in this article,. However, as pointed out, loco-regional administration of radiation nanomedicines is a promising route that overcomes these limitations. Interestingly, a first-in-human clinical trial of ^188^Re-labeled nanoliposomes for local treatment of GBM administered by CED has been launched (ClinicalTrials.gov identifier: NCT01906385). SPECT imaging of patients in this trial demonstrated retention of ^188^Re-labeled nanoliposomes at the infusion site in the brain with no redistribution to other regions of the brain (Fig. 10) (Woodall et al. 2021) These imaging results in patients with GBM administered ^188^Re-labeled nanoliposomes by CED are very similar to those that we reported for [^177^Lu]Lu-MCP-AuNPs infused by CED in NRG mice with orthotopic human GBM tumours (Fig. 9b) (Georgiou et al. 2023).

#### Regulatory guidelines for nanomedicines

To our knowledge, there are no specific regulatory guidelines for radiation nanomedicines. However, guidelines for nanomedicines have been published by the U.S. FDA (Paradise 2019; Thapa and Kim 2023) and the European Medicines Agency (EMA) and Health Canada (Dri et al. 2023). The quality requirements for nanomedicines vary between regulatory agencies, but generally include evaluation of the physico-chemical properties of the NPs such as particle size and morphology, stability, surface charge, drug loading and release, sterility and endotoxins testing and toxicity assessment (Anonymous 2022; Dri et al. 2023). Radiation nanomedicines would require additional tests for measurement of radiochemical purity (RCP) and radionuclide purity (RNP), stability against loss of radiometal in serum, organ dosimetry and radiation toxicity. Good Manufacturing Practices (GMP) for radiation nanomedicines remain to be established and are beyond the scope of this article. Nonetheless, Hernandez-Jimenez et al. (2022) reported production of ^177^Lu[Lu]-iFAP/iPSMA NPs (23 nm) under GMP conditions in an ISO Class 5 clean air environment. Quality control testing included measurement of RCP and RNP, particle size, surface functionalization, heavy metal content, carrier lutetium quantification, and sterility and endotoxins tests. Finally, there is the possibility that radiation nanomedicines packaged and retained in an implantable delivery system, e.g. a NPD incorporating radiolabeled AuNPs as previously reported by our group (Cai et al. 2022), may be considered brachytherapy and regulated as a medical device (Beers et al. 2019).

## Conclusions

Radiation nanomedicines have shown great promise for treating cancer in preclinical animal tumour models. Local intratumoural administration avoids sequestration of NPs by the liver and spleen, depositing high absorbed doses in tumours and low doses in normal organs. This provides high effectiveness for treating tumours, while minimizing normal tissue toxicity. Opportunities to locoregionally deliver radiation nanomedicines to cancers, particularly for tumours that are anatomically restricted should be explored, based on the highly encouraging results obtained to date for local cancer treatment in preclinical animal tumour models. Future research opportunities in the field of radiation nanomedicines include optimizing the design of NPs to minimize liver and spleen uptake after i.v. injection, development of new radiochemistry to incorporate therapeutic radionuclides into NPs, strategies to actively transport NPs to tumours by conjugating targeting ligands, and studies of radiation nanomedicines combined with checkpoint immunotherapy to promote an immune-mediated cytotoxic T-cell response to amplify and extend their therapeutic effects.

## Data Availability

Not applicable.

## Abbreviations

5-FU: 5-Fluorouracil
AE: Auger electrons
ALT: Alanine aminotransferase
AuNCs: Gold nanoclusters
AuNPs: Gold nanoparticles
AuNRs: Gold nanorods
BC: Breast cancer
BCM: Block copolymer micelles
BED: Biologically effective dose
BLI: Bioluminescence
BMEDA: *N*,*N*-Bis(2-Mercaptoethyl)-*N*′,*N*′-Diethylethylenediamine
CBC: Complete blood cell
CCS: Canadian cancer society
CE: Internal conversion electrons
CED: Convection enhanced delivery
CEPA: 3-Phosphonopropionic acid
CHO: Chinese hamster ovary
CHX-DTPA: Cyclohexane-1,2-diethylenetriaminepentaacetic acid
cis-Pt: Cisplatin
cMORF: Complementary morpholino oligonucleotides
CNCs: Cellulose nanocrystals
CNT: Carbon nanotubes
Cr: Creatinine
CREATE: Collaborative research and training experience
cRGD: Cyclic arginine-glycine-aspartic acid
CS: Clonogenic survival
CSC: Cancer stem cell
CT: Computed tomography
CXCL12: C-X-C motif chemokine 12
CXCR4: C-X-C chemokine receptor type 4
Cys: Cysteine
D_10_: Dose required to decrease the surviving fraction of cells to 0.10
DAMP: Damage-associated molecular patterns
DARP: Designed ankyrin repeat protein
DEF: Dose-enhancement factor
DFO: Desferrioxamine
DLS: Dynamic light scattering
DMSA: Dimercaptosuccinic acid
DOTA: 1,4,7,10-Tetraazacyclododecane-*N*,*N*′,*N*′′,*N*′′′-tetraacetic acid
Dox: Doxorubicin
DPK: Dose-point kernel
DRT: Dual receptor-targeted
DSB: Double stranded breaks
DTPA: Diethylenetriamine pentaacetate
DTT: Dithiothreitol
DVH: Dose-volume histograms
DXR: Doxorubicin
EC: Electron capture
EGF: Epidermal growth factor
EGFR: Epidermal growth factor receptor
EMA: European medicines agency
EPR: Enhanced permeability and retention
EUD: Effective uniform dose
EUD: Equivalent uniform dose
FAP: Fibroblast activation protein
FDA: Food and drug administration
FDG: Fluorodeoxyglucose
FR-α: Folate receptor alpha
G4 PAMAM: Generation 4 poly(amidoamine) dendrimer
GBM: Glioblastoma multiforme
G-CSF: Granulocyte colony-stimulating factor
GIP: Glucose-dependent insulinotropic polypeptide
G_MI_: Monosialoganglioside
GMP: Good manufacturing practices
GRP: Gastrin releasing peptide
GRPR: Gastrin releasing peptide receptor
GSH: Glutathione
HER2: Human epidermal growth factor receptor-2
HNSCC: Head and neck squamous cell carcinoma
HYNIC: Hydrazinonicotinic acid
i.p.: Intraperitoneal
ISO: International organization for standardization
i.t.: Intratumoral
i.v.: Intravenous
ID: Injected dose
iFAP: Fibroblast activation protein inhibitor
IgG: Immunoglobulin G
IONPs: Iron oxide nanoparticles
iPSMA: Prostate-specific membrane antigen inhibitor
KLH: Keyhole limpet hemocyanin
LA: Lipoic acid
LD_50_: Lethal dose-50%
LET: Linear energy transfer
Lipo-Dox: Liposomal doxorubicin
LN: Lymph node
LNC: Lipid nanocapsules
MC: Monte carlo
MCF: Mangiferin
MCNP: Monte carlo N-particle software
MCP: Metal-chelating polymers
MIONP: Magnetic iron oxide nanoparticles
MIRD: Medical internal radiation dose
MLV: Multi-lamellar vesicles
MMP: Matrix metalloproteinases
MPS: Mononuclear phagocyte system
MRI: Magnetic resonance imaging
MTD: Maximum tolerated dose
MTX: Methotrexate
MUVEL: Multivesicular liposomes
Nano-RER: Nano-radio enhancement ratio
^Nat^Lu_2_O_3_: Natural lutetium oxide
NaA: Zeolite A sodium
NaYF_4_: Sodium yttrium fluoride
NCs: Nanocapsules
NDEF: Nuclear dose enhancement factor
NHS: N-hydroxysuccinimide
nIR: Near infrared
NLS: Nuclear translocation sequence
NMRI: Naval medical research institute
NOD/SCID: Non-obese diabetic/severe combined immunodeficiency disease
NP: Nanoparticles
NPD: Nanoparticle depot
NRG: NOD-Rag2-IL2rγTm1/Rj
OPSS: Orthopyridyldisulphide
p.i.: Post-injection
PBS: Phosphate buffered saline
PCL: Poly-caprolactone
PD-L1: Programmed cell death ligand-1
PE: Photoelectrons
PE40: Pseudomonas exotoxin
PEG: Poly ethelyne glycol
PET: Positron emission tomography
POND: Polymer nanoparticles for drug delivery
p-SCN-Bn-DTPA: S-2-(4-Isothiocyanatobenzyl)-diethylenetriamine pentaacetic acid
PSMA: Prostate specific membrane antigen
PTT: Photothermal therapy
RBE: Radiobiological effectiveness
RCP: Radiochemical purity
RER: Radio enhancement ratio
RGD: Arginine-glycine-aspartic acid peptide
RNP: Radionuclide purity
ROS: Reactive oxygen species
s.c.: Subcutaneous
SA: Specific activity
SCN: Benzyl isothiocyanate
SPECT: Single photon emission computed tomography
SPION: Superparamagnetic iron oxide nanoparticles
SPN: Semiconducting polymer nanoparticles
SRT: Single-receptor targeted
SUV: Small unilamellar vesicles
SVA: Hydroxysuccinimide valerate
SWCNT: Single walled carbon nanotubes
Tat: Transactivation of transcription
VEGF: Vascular endothelial growth factor
WBC: White blood cell
XRT: X-radiation therapy
α-MSH: α-Melanocyte stimulating hormone
γH2AX: Phosphorylated histone-2AX
